# The ReIMAGINE prostate cancer risk study protocol: A prospective cohort study in men with a suspicion of prostate cancer who are referred onto an MRI-based diagnostic pathway with donation of tissue, blood and urine for biomarker analyses.

**DOI:** 10.1371/journal.pone.0259672

**Published:** 2022-02-24

**Authors:** Teresa Marsden, Neil McCartan, Louise Brown, Manuel Rodriguez-Justo, Tom Syer, Giorgio Brembilla, Mieke Van Hemelrijck, Ton Coolen, Gerhardt Attard, Shonit Punwani, Caroline M. Moore, Hashim U. Ahmed, Mark Emberton, on behalf of the ReIMAGINE Study Group

**Affiliations:** 1 UCL Division of Surgical & Interventional Sciences, University College London, London, United Kingdom; 2 Department of Urology, University College London Hospitals NHS Foundation Trust, London, United Kingdom; 3 MRC Clinical Trials Unit, University College London, London, United Kingdom; 4 Research Department of Pathology, University College London, London, United Kingdom; 5 Department of Pathology, University College London Hospitals NHS Foundation Trust, London, United Kingdom; 6 Centre for Medical Imaging, University College London, London, United Kingdom; 7 School of Cancer and Pharmaceutical Sciences, Kings College London, London, United Kingdom; 8 London Institute for Mathematical Sciences, London, United Kingdom; 9 Cancer Institute, University College London, London, United Kingdom; 10 Imperial Prostate, Division of Surgery, Department of Surgery and Cancer, Faculty of Medicine, Imperial College London, London, United Kingdom; 11 Imperial Urology, Imperial College Healthcare NHS Trust, London, United Kingdom; Australian National University College of Health and Medicine, AUSTRALIA

## Abstract

**Introduction:**

The ReIMAGINE Consortium was conceived to develop risk-stratification models that might incorporate the full range of novel prostate cancer (PCa) diagnostics (both commercial and academic).

**Methods:**

ReIMAGINE Risk is an ethics approved (19/LO/1128) multicentre, prospective, observational cohort study which will recruit 1000 treatment-naive men undergoing a multi-parametric MRI (mpMRI) due to an elevated PSA (≤20ng/ml) or abnormal prostate examination who subsequently had a suspicious mpMRI (score≥3, stage ≤T3bN0M0). Primary outcomes include the detection of ≥Gleason 7 PCa at baseline and time to clinical progression, metastasis and death. Baseline blood, urine, and biopsy cores for fresh prostate tissue samples (2 targeted and 1 non-targeted) will be biobanked for future analysis. High-resolution scanning of pathology whole-slide imaging and MRI-DICOM images will be collected. Consortium partners will be granted access to data and biobanks to develop and validate biomarkers using correlation to mpMRI, biopsy-based disease status and long-term clinical outcomes.

**Results:**

Recruitment began in September 2019(n = 533). A first site opened in September 2019 (n = 296), a second in November 2019 (n = 210) and a third in December 2020 (n = 27). Acceptance to the study has been 65% and a mean of 36.5ml(SD+/-10.0), 12.9ml(SD+/-3.7) and 2.8ml(SD+/-0.7) urine, plasma and serum donated for research, respectively. There are currently 4 academic and 15 commercial partners spanning imaging (~9 radiomics, artificial intelligence/machine learning), fluidic (~3 blood-based and ~2urine-based) and tissue-based (~1) biomarkers.

**Conclusion:**

The consortium will develop, or adjust, risk models for PCa, and provide a platform for evaluating the role of novel diagnostics in the era of pre-biopsy MRI and targeted biopsy.

## 1 Introduction

The prostate cancer (PCa) diagnostic pathway has undergone significant change in recent years and a number of guidelines now recommend multi-parametric MRI as the first line investigation for those with suspected organ-confined disease [1–3]. Before this, diagnosis and risk stratification was reliant upon clinical examination, PSA and systematic transrectal ultrasound-guided (TRUS) biopsy, but we are now aware how imprecise this diagnostic strategy was. Recent studies that used an exacting reference standard showed that over half of men with clinically significant prostate cancer were incorrectly diagnosed when exposed to TRUS-biopsy [4].

The incorporation of imaging into the diagnostic pathway has nearly doubled the probability of accurate baseline risk stratification compared to what went before. This is because biopsy sampling is now directed to areas of radiologically abnormal tissue with a high probability of clinically significant PCa rather than reliance on a systematic sampling approach which is arguably a somewhat random process [4]. The result is fewer missed clinically significant cancers, less over-diagnosis of clinically insignificant PCa and less harm from fewer men biopsied.

### 1.1 Aims and objectives

Current prostate cancer risk models (largely in the form of risk calculators) continue to inform treatment allocation but are based upon data collected during an era in which systematic TRUS-biopsy was the standard of care. When the histological outputs of modern-day diagnostics are applied to such models there is a danger of risk inflation as ‘risk’ is amplified (towards the truth). The result is that mens’ expected risk might be exaggerated and the risk of over-treatment increases as a result [5, 6].

The ReIMAGINE consortium was conceived to address the need for new or adjusted prostate cancer risk stratification models to reflect MRI-directed diagnostic strategy. The ReIMAGINE consortium is a collaboration between several academic and commercial partners to use diagnostics that span imaging, fluidic and tissue-based data to define novel, measurable and deeply phenotyped mpMRI disease cohorts, known as endotypes. Within the ReIMAGINE Prostate Cancer Risk study, the principal work-strand of the ReIMAGINE Consortium, inputs collected during MR-led diagnostics will be used to inform novel baseline risk-stratification systems and prognostic models.

The longitudinal component of the study will assess cancer progression, time to metastasis and prostate cancer related death through national healthcare data linkage. Deep phenotyping of measurable mpMRI disease endotypes and long-term follow up will permit the first insights into characteristics that define significant disease and redefine risk stratification in the mpMRI diagnostic era.

## 2 Design and methods: The ReIMAGINE prostate cancer risk protocol

### 2.1 Study design

ReIMAGINE Prostate Cancer Risk is a multi-centre, prospective, observational, longitudinal cohort study of patients referred to secondary care with a suspicion of prostate cancer (elevated PSA and/or abnormal digital rectal exam) or those who are having further tests for re-stratification of pre-existing low risk disease. The study represents level 1b evidence for diagnostic and prognostic studies [7]. Participants will be recruited from a number of high-volume NHS prostate cancer diagnostic centres in London, U.K. where pre-biopsy mpMRI followed by MRI-targeted prostate biopsies are established as standard of care.

Consenting men will be asked to donate research samples (blood, urine and fresh prostate tissue) for biomarker analysis at the time of their NHS standard of care prostate biopsies. Consent will also be sought for research access to pre-biopsy prostate mpMRI data and national medical records until death (should further funding be secured).

Biomarker analysis of donated samples will complete the cross-sectional component of the study. Participants exit the study following collection of tissue samples and revert to the NHS standard of care prostate cancer pathway. Standard of care pathology results are provided during a routine NHS clinic in accordance with local hospital protocol.

This manuscript reflects the current protocol version 2.1, 12 November 2019.

### 2.2 Patient and public involvement (PPI)

The ReIMAGINE project includes a work strand which focuses solely on patient and public involvement (PPI). The PPI committee consists of patients, and the wider public, who have been affected by, or have experience of, prostate cancer. The committee meet at least once every three months to inform prioritisation of research outputs, steer media outputs, and assist with development of study materials e.g. participant information videos. All patient facing documents are reviewed by a PPI representative or Chair of the PPI Sub-Committee, and its members.

The PPI team has been responsible for the development of the ReIMAGINE Consortium website (https://www.reimagine-pca.org). The website hosts a range of patient and media information resources relevant to each work strand of the consortium. Resources include details of the consortium directors, consortium partners, and links to participant and public information videos. Additional COVID-19 specific resources have been provided on the website since the study resumed recruitment in April 2020, and outline the steps taken to reduce the risk of COVID-19 exposure during a study visit.

### 2.3 Patient population

**Fig 1:**
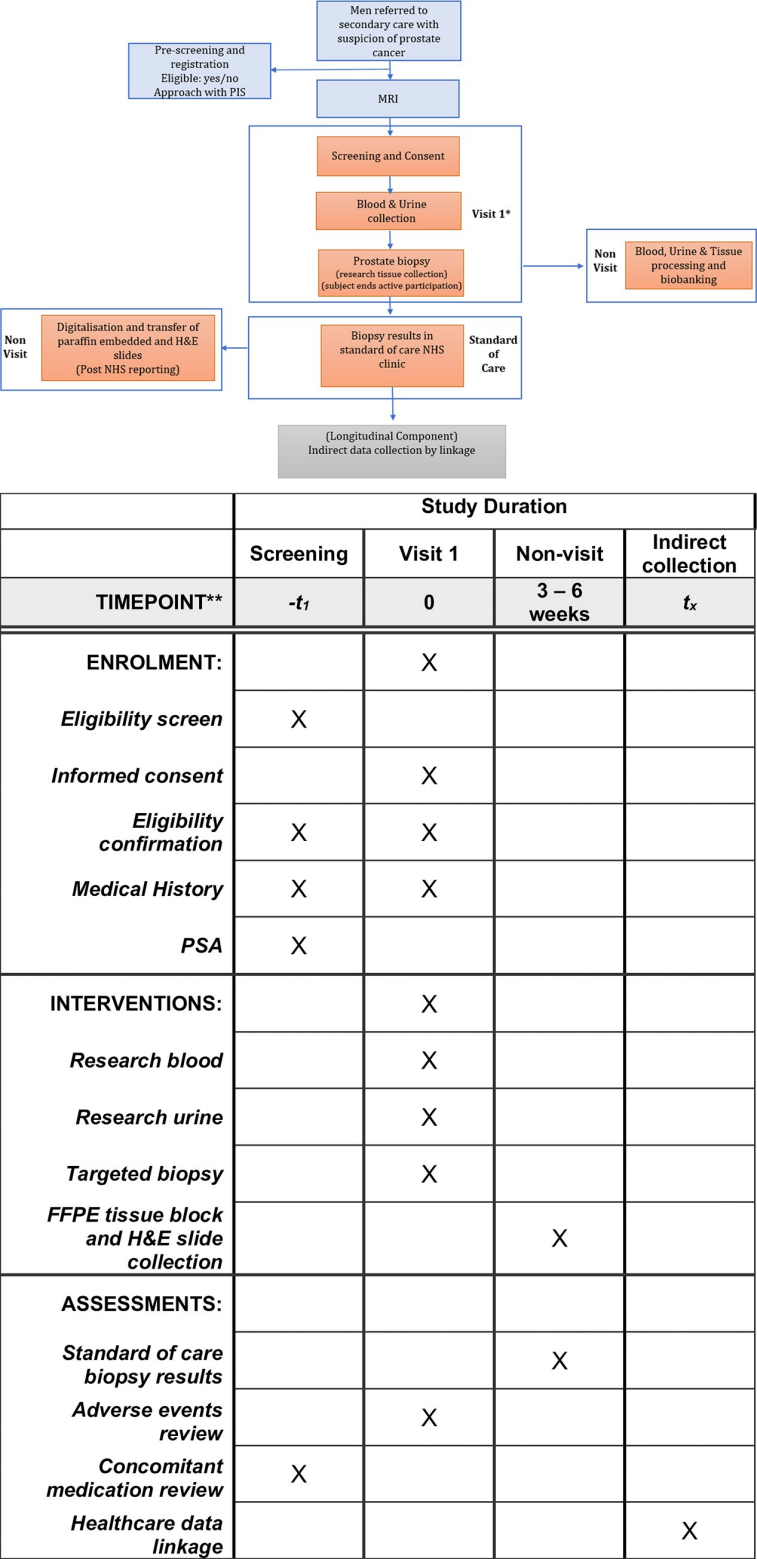
**a.** ReIMAGINE Prostate Cancer Risk Study Design. ReIMAGINE Prostate Cancer Risk is a multi-centre, prospective, observational, longitudinal cohort study of patients referred to secondary care with a suspicion of prostate cancer (elevated PSA and/or abnormal digital rectal exam) or those who are having further tests for re-stratification of pre-existing low risk disease. Consenting men will be asked to donate research samples (blood, urine and fresh prostate tissue) for biomarker analysis at the time of their NHS standard of care prostate biopsies (Visit 1). Consent will also be sought for research access to pre-biopsy prostate mpMRI data and national medical records until death (should further funding be secured). Biomarker analysis of donated samples will complete the cross-sectional component of the study (both academic and commercial). Participants exit the study following collection of tissue samples and revert to the NHS standard of care prostate cancer pathway. Standard of care pathology results are provided during a routine NHS clinic in accordance with local hospital protocol. Access to standard of care diagnostic biopsy tissue will be sought once the participating centre has completed all necessary diagnostic evaluation. Formalin-fixed paraffin-embedded (FFPE) tissue blocks and haematoxylin and eosin (H&E) stained slides will be requested by ReIMAGINE staff and anonymised H&E slides will be scanned using high resolution scanners and uploaded to the ReIMAGINE digital pathology database. Consent will be sought to obtain long-term patient outcomes from national health records, such as the Office for National Statistics, NHS Digital, Public Health England and other applicable NHS information systems or national databases. Initially, data will be collected for three years after the last patient visit with the intention to collect data up to participant death once adequate funding is secured. This will complete the longitudinal component of the study. **b.** ReIMAGINE Prostate Cancer Risk Study SPIRIT schedule of enrolment, interventions, and assessments.

ReIMAGINE Risk aims to recruit 1000 men who are undergoing standard of care mpMRI directed biopsy following detection of an MRI lesion. Men with a PSA of less than or equal to 20 ng/ml who have undergone an mpMRI prostate during an NHS diagnostic work-up will be invited to participate in the study if a lesion scoring 3, 4 or 5 (radiological stage </ = T3b; clinical or radiological stage N0 and M0) is identified on mpMRI and they have accepted the need for a targeted and systematic biopsy (Fig 1). Either Likert or PIRADSv2.1 scoring will be acceptable. There must be no history of prior prostate cancer treatment (chemical, biological, ablative, surgical, radiotherapy) and no anti-androgen exposure in the preceding 6 months (5-alpha reductase inhibitors permitted). Full inclusion and exclusion criteria are outlined in Table 1.

**Table 1:** ReIMAGINE risk study inclusion and exclusion criteria.

**Inclusion Criteria**
Any man with a PSA 20ng/ml or less (value recorded <90 days before study entry)
Men who have undergone a prostate mpMRI as a standard NHS diagnostic work-up
mpMRI lesion (or area of suspicion) conforming to Likert / PIRADSv2.1 3, 4 or 5 undergoing a targeted and systematic transperineal biopsy
Radiological stage T3b or less
Clinical or radiological stage N0 and M0
No anti-androgen exposure in the preceding 6 months (5-alpha reductase inhibitors permitted)
No prior treatment for prostate cancer (chemical, biological, ablative, surgical, radiotherapy). Prior ADT permitted with adequate wash-out as described above (>6months)
Previous TURP is permitted
Willing and able to provide written informed consent
**Exclusion Criteria**
Men unable to donate tissue, blood or urine
Previous prostate cancer treatment
Previous prostate biopsy <12 months from date of the mpMRI scan used to assess study eligibility (scoring Likert / PIRADSv2.1 3, 4 or 5)

ReIMAGINE Prostate Cancer Risk is a multi-centre, prospective, observational, longitudinal cohort study of patients referred to secondary care with a suspicion of prostate cancer (elevated PSA and/or abnormal digital rectal exam) or those who are having further tests for re-stratification of pre-existing low risk disease. Men with a PSA of less than or equal to 20 ng/ml who have undergone an mpMRI prostate during an NHS diagnostic work-up will be invited to participate in the study if a lesion scoring 3, 4 or 5 (radiological stage </ = T3b; clinical or radiological stage N0 and M0) is identified on mpMRI and they have accepted the need for a targeted and systematic biopsy (Fig 1). Either Likert or PIRADSv2.1 scoring will be acceptable. There must be no history of prior prostate cancer treatment (chemical, biological, ablative, surgical, radiotherapy) and no anti-androgen exposure in the preceding 6 months (5-alpha reductase inhibitors permitted). Full inclusion and exclusion criteria are outlined in Table 1.

The SPIRIT schedule of enrolment, interventions, and assessments is provided to outline the aforementioned study design.

### 2.4 Exposure variable

The main exposure variable is the deep phenotyping of the MRI-visible lesion. This will be achieved using additional targeted sampling of MR-identifiable lesions using either visual-estimation or image-fusion software, dependent on standard approach at each recruiting centre. As such, underlying inter-tumoral heterogeneity will be captured [8, 9]. A control sample collected from an area of unremarkable tissue on MRI (Likert / PIRADSv2.1 score 1 or 2) will further inform the characterisation of MR lesions.

### 2.5 Phase of study

ReIMAGINE Prostate Cancer Risk is currently recruiting at three London sites. The first site opened to recruitment in September 2019, a second centre in November 2019 and a third in December 2020. Recruitment was briefly halted between March and April 2020 due to the COVID-19 pandemic.

So far, 533 participants have been recruited across three sites. Recruitment is expected to complete in March 2022.

Acceptance to the study has been 65% and a mean of 36.5ml (SD+/-10.0), 12.9ml (SD+/-3.7) and 2.8ml (SD+/-0.7) urine, plasma and serum donated for research, respectively.

## 3 Study schedule

### 3.1 Screening and recruitment

Potential participants will be identified at the time of referral to a participating NHS centre, during a standard of care urology outpatient appointment or during screening of cancer waiting lists and theatre diaries. Participants will be approached in the first instance by either clinical staff or ReIMAGINE study staff (clinical trial practitioners). The first contact with eligible participants may be a telephone call or an invitation letter ahead of a standard of care prostate biopsy. Participating NHS centres may run a prostate cancer “one-stop” clinic whereby suspected cancer patients undergo biopsy on the day of baseline diagnostic mpMRI. Therefore, contact by telephone ahead of the visit is essential to maximise accrual. Potential participants will be offered a patient information leaflet to be sent electronically or by post ahead of their biopsy. All those approached or considered for recruitment will be recorded on the screening log.

During screening, and at the time of consent, patients will be assessed against the inclusion and exclusion criteria outlined in Table 1. Once eligibility is confirmed, written informed consent will be sought. Eligible patients will be invited to consent to donation of blood, urine and three prostate tissue cores for biomarker analysis (to be collected at the time of standard of care biopsy, therefore no additional visits beyond the standard of care are required). Participants will also consent to donation of pseudonymised pre-biopsy mpMRI files.

Consent to access standard of care biopsy formalin-fixed paraffin-embedded (FFPE) tissue blocks and haematoxylin and eosin (H&E) slides will be requested once the holding NHS centre has fulfilled all diagnostic requirements. Agreement will also be sought for healthcare data linkage through NHS digital, Public Health England and other relevant bodies to inform longitudinal outcomes.

Recruiting sites will register fully eligible and consenting patients on a research database (Research Data Collection Service, REDCap) hosted securely by UCL. Project data will be periodcially exported, after additional de-identification, from REDCap to a central integrated database (the ReIMAGINE Clinical Data Lake, CDL) hosted by Philips in the European Union (EU). Each participant will be allocated a unique study identification number. Identifiable data will be held on a separate REDCap database hosted only by UCL and used during the evaluation of longitudinal outcomes.

#### 3.1.1 Visit 1

**Fig 2:**
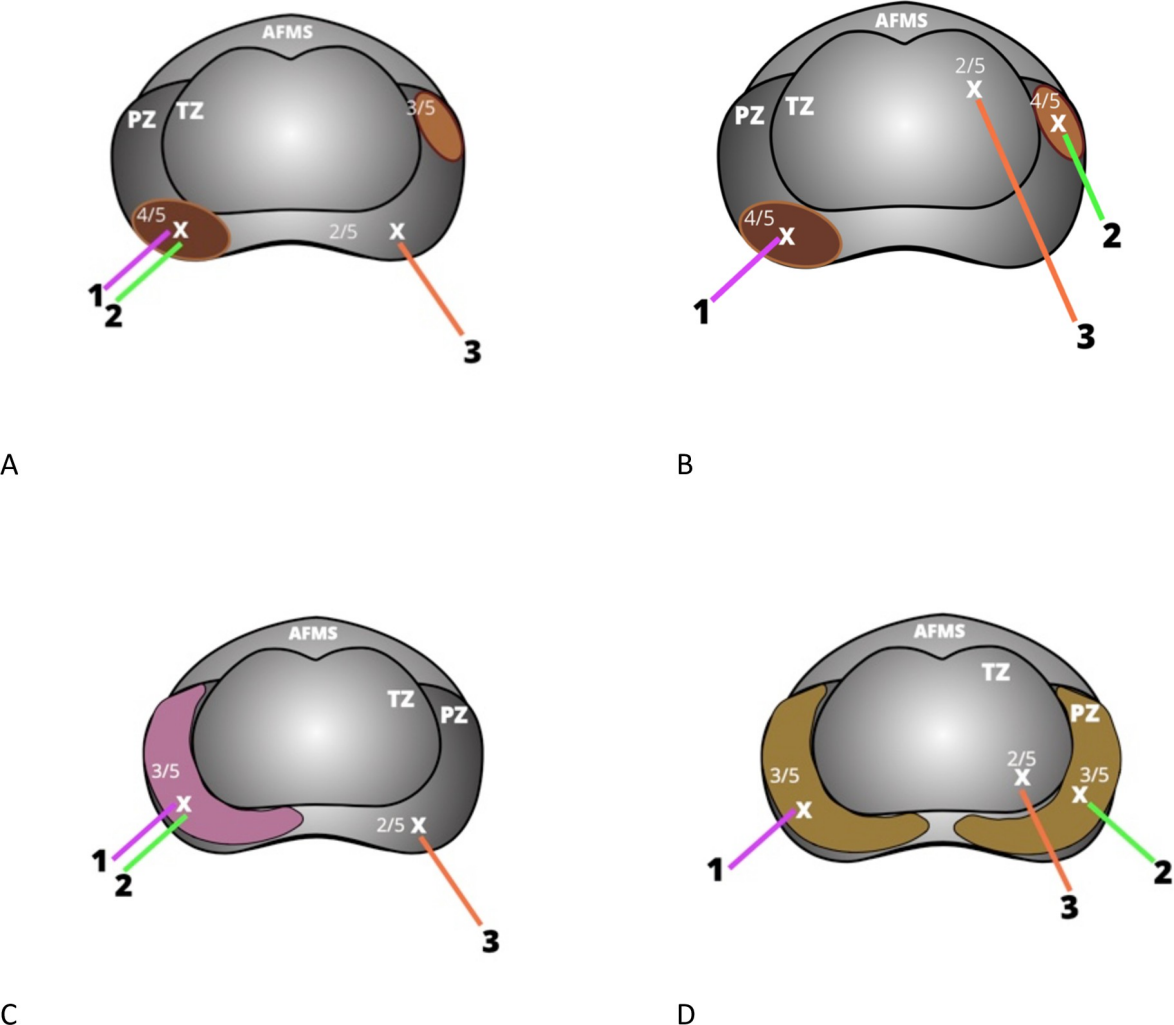
Location of research prostate tissue cores in ReIMAGINE prostate cancer risk. A) Cores 1 and 2 are collected from the centre of the highest scoring lesion (denoted as Likert / PIRADSv2.1 score 4 in this figure) if there are no other lesions of the same score. The area of Likert / PIRADSv2.1 score 3 tissue in the left peripheral zone is not sampled for research. Core 3 is collected from an area of Likert / PIRADSv2.1 score 1 or 2 tissue (denoted as Likert / PIRADSv2.1 score 2 tissue in this figure). B) Where there is more than one lesion of the highest score, Core 1 is collected from the centre of the highest scoring lesion with greatest volume and Core 2 from the centre of the next largest lesion with the same score (both denoted as Likert / PIRADSv2.1 score 4 PZ lesions in this figure). Core 3 is collected from an area of Likert / PIRADSv2.1 score 1 or 2 tissue. C) If there is diffuse Likert / PIRADSv2.1 score 3 change to one peripheral zone (PZ) the cores should be collected from the centre of the area of score 3 change denoted on the radiology report (Cores 1 and 2). The third research core will be collected from an area of non-suspicious (Likert / PIRADSv2.1 score 1 or 2) tissue. D) If there is diffuse Likert / PIRADSv2.1 score 3 to the PZs bilaterally, the side of greater suspicion should be sampled first (Core 1), and a second core collected from the contralateral score 3 PZ (Core 2). The third research core will be collected from an area of non-suspicious (Likert / PIRADSv2.1 score 1 or 2) tissue.

Following written informed consent, participant demographics, height, weight, medical history and medication history will be recorded. Biological samples (donated blood and first pass / early morning urine) will be collected on the day of standard of care biopsy. Three additional research core biopsies (two from the primary region of radiological interest and one control biopsy from an area of non-suspicious Likert / PIRADSv2.1 1 or 2 tissue within the contralateral lobe of the prostate will be collected at the time of standard of care diagnostic biopsies (Fig 2). Samples will be processed and stored at a UCL laboratory until recruitment of all study participants is complete. Details of sample processing and storage are outlined in section 4.0. Once recruitment is complete, samples will be distributed to academic and commercial partners for analysis.

#### 3.1.2 Non-visit

Access to standard of care diagnostic biopsy tissue will be sought once the participating centre has completed all necessary diagnostic evaluation. Formalin-fixed paraffin-embedded (FFPE) tissue blocks and haematoxylin and eosin (H&E) stained slides will be requested by ReIMAGINE staff and anonymised H&E slides will be scanned using high resolution scanners and uploaded to the ReIMAGINE digital pathology database. Slices from standard of care FFPE tissue blocks will be collected in line with study standard operating procedures before returning all slides and blocks to the relevant NHS pathology department.

#### 3.1.3 Analyses

Blood components (white blood cells, plasma, serum) urine and tissue will undergo molecular analysis including genomic, mRNA expression and protein studies by academic and industry consortium partners. These will include, but are not limited to, next-generation sequencing, whole-genome micro-array expression profiling, immunohistochemistry and immunofluorescence and ELISA tests.

#### 3.1.4 Long-term healthcare data linkage

Consent will be sought to obtain long-term patient outcomes from national health records, such as the Office for National Statistics, NHS Digital, Public Health England and other applicable NHS information systems or national databases. Initially, data will be collected for three years after the last patient visit with the intention to collect data up to participant death once adequate funding is secured. This will complete the longitudinal component of the study.

### 3.2 Definition of end of study

Following the collection of research samples (blood, urine and prostate tissue) men will revert to the NHS standard of care prostate cancer pathway at their centre. No further study visits are required. Men will undergo study follow up for 4 to 8 weeks after the biopsy for pathology results to be collected. Once complete, active data collection for the cross-sectional component of the study is complete. Longitudinal data collection through health data linkage will continue for three years and until death if adequate funding is secured.

## 4 Study interventions

ReIMAGINE prostate cancer risk will provide a platform for commercial and academic partners to run and calibrate novel biomarker tests within a cohort of suspected prostate cancer patients undergoing mpMRI targeted prostate biopsy. Once recruitment and sample collection is complete, specimens and pre-biopsy mpMRI data will be distributed to partner academic and commercial teams for analysis.

### 4.1 mpMRI databank

Multiparametric MR imaging will be performed as NHS standard of care [1, 10]. Reporting will be according to study SOPs and include Likert and PIRADSv2.1 scales documented within a structured electronic case report form (eCRF). Lesions, and the prostate itself, will be contoured and segmented prior to upload to the study image repository. Details of image processing and storage are outlined in Appendix I in S1 File.

### 4.2 Blood sample collection

Between 50 and 100 ml of blood will be collected from each consenting study participant by a trained phlebotomist or ReIMAGINE Clinical Trial Practitioner during visit 1. Venepuncture and sample collection will be performed in accordance with the ReIMAGINE Risk Blood Sampling SOP. The required samples are outlined in Table 2. A minimum blood donation of 50 ml is requested (up to a maximum of 100 ml). Whole blood samples will be processed by a Laboratory Technician or Clinical Trial Practitioner at a project-affiliated laboratory or an allocated laboratory area within the hospital site. Details of sample processing are outlined in Appendix II in S2 File.

**Table 2:** Blood samples collected during the ReIMAGINE risk study.

Order of draw (by collection tube)	Number of tubes to be collected	Volume per tube (ml)
EDTA	1	10
SST	1	8.5
Streck^TM^	1	10
EDTA	1	10
PAXgene® Blood RNA	1	2.5
EDTA	1	10
Extra EDTA (optional)	0–6	10

Between 50 and 100 ml of blood will be collected from each consenting study participant by a trained phlebotomist or ReIMAGINE Clinical Trial Practitioner during visit 1. Venepuncture and sample collection will be performed in accordance with the ReIMAGINE Risk Blood Sampling SOP. The required samples and order of draw is outlined in Table 2.

Any sample processed at clinical sites will be temporarily stored onsite at -80°C. Following initial onsite processing, pseudonymised samples will be logged onto the study FreezerPro database. Samples will be transferred by courier to a study-affiliated UCL laboratory at regular intervals and stored at -80°C.

### 4.3 Urine sample collection

A first pass or early morning urine sample will be collected in line with the study SOP by study practitioners during visit 1. Urine collection will always take place prior to prostate biopsy. The pseudonymised samples will be stored temporarily at local sites, transferred to a study-affiliated laboratory and logged within the study FreeserPro database. Both unspun and centrifuged urine will be stored within the study biobank. Spun samples will be centrifuged using a one-step process (3300g for 20 mins, with a break, at room temperature). Both centrifuged and unspun urine will be stored at -80°C as will the residual cell pellet.

### 4.4 Prostate tissue collection

Consenting patients will donate three additional cores of prostate tissue at the time of their standard of care prostate biopsy (Fig 2). We do not anticipate that collection of three additional tissue cores will increase the likelihood of side effects or adverse events relative to standard of care biopsy alone. The risks and adverse outcomes of prostate biopsy quoted within the ReIMAGINE Risk patient information sheet are outlined in Table 3. Research cores will be targeted (visual-estimation or software fusion) to areas of interest identified on pre-biopsy mpMRI. Research cores will only be attempted after all required standard of care diagnostic biopsies have been collected.

**Table 3:** Side effect profile of a targeted transperineal prostate biopsy as stated in the ReIMGAINE prostate cancer risk study patient information sheet.

Side effect	Proportion of men	Duration
Pain/discomfort in back passage	Almost all	Temporary for 1–2 days
Burning when passing urine	Almost all men	Self-resolving, 1–3 days
Bloody Urine	Almost all	Self-resolving, 1–7 days
Bloody Sperm	Almost all	Lasting up to 3 months
Poor erections	1–2 in 100	Temporary for 1–6 weeks
Infection of skin/urine	1–2 in 100	7 days with treatment with antibiotics
Infection of skin/urine needing admission and intravenous antibiotics	Less than 1 in 500	7–14 days with treatment with antibiotics. Rarely up to 28 days of antibiotics after leaving hospital.
Difficulty passing urine requiring catheter placement for up to a week. A catheter is a soft plastic tube placed into the bladder to drain urine.	1 in 100	3–7 days
Bruising of skin	Almost all	Self-resolving, 7–14 days
Bruising spread to scrotum	1 in 100	Self-resolving, 7–28 days

Eligible patients at recruiting sites will be provided with a study information leaflet at least 24 hours prior to consenting to study entry. The risk profile of targeted transperineal prostate biopsies is included in the study patient information leaflet and is shown in this figure.

All cores will be collected in line with the study Tissue Collection SOP. The first research core will be collected from the centre of the lesion with the highest Likert / PIRADSv2.1 score and greatest volume (lesion 1). The second sample will be collected from the centre of lesion 1 unless multiple lesions with the same Likert / PIRADSv2.1 score exist.

If multiple lesions with the same Likert / PIRADSv2.1 score exist, the second core should be collected from the next largest lesion with the same MRI score. Lesion volume will be determined by mpMRI reporting or MRI-US fusion software. If there is diffuse Likert / PIRADSv2.1 score 3 change to one peripheral zone (PZ) the core should be collected from the centre of the area denoted on the radiology report. If there is diffuse Likert / PIRADSv2.1 score 3 to the PZs bilaterally, the side of greater suspicion should be sampled first. Patients exhibiting diffuse change to the whole prostate should not be recruited.

The third research core will be collected from an area of non-suspicious (Likert / PIRADSv2.1 score 1 or 2) tissue. All biopsy cores should be collected from the centre of each targeted lesion. Table 4 summarises the location from which to collect each prostate biopsy core.

**Table 4:** Prostate tissue sampling locations for the ReIMAGINE prostate cancer risk study.

	Lesion to target	Area within lesion to target
**Core 1**	Lesion with the highest Likert / PIRADSv2.1 score and greatest volume (lesion 1).	Centre of lesion 1.
		If there is diffuse change throughout the PZs/TZs uni- or bi-laterally, target the centre of the area of greatest suspicion as defined by the reporting radiologist.
**Core 2**	Lesion 1, unless multiple lesions with the same Likert / PIRADSv2.1 score exist.	Centre of lesion 1 or centre of lesion 2 if multiple lesions with the same Likert / PIRADSv2.1 score exist.
	In which case, target the next largest lesion with the same Likert / PIRADSv2.1 score (lesion 2).	
**Core 3**	Collect from an area of non-suspicious (Likert / PIRADSv2.1 score 1 or 2) tissue.	Any non-suspicious area e.g. contralateral transition zone (TZ).

The first research core will be collected from the centre of the lesion with the highest Likert / PIRADSv2.1 score and greatest volume (lesion 1). The second sample will be collected from the centre of lesion 1 unless multiple lesions with the same Likert / PIRADSv2.1 score exist. If multiple lesions with the same Likert / PIRADSv2.1 score exist, the second core should be collected from the next largest lesion with the same MRI score. The third and final research core will be collected from an area of radiologically normal tissue (defined as Likert / PIRADSv2.1 1 or 2).

**Fig 3:**
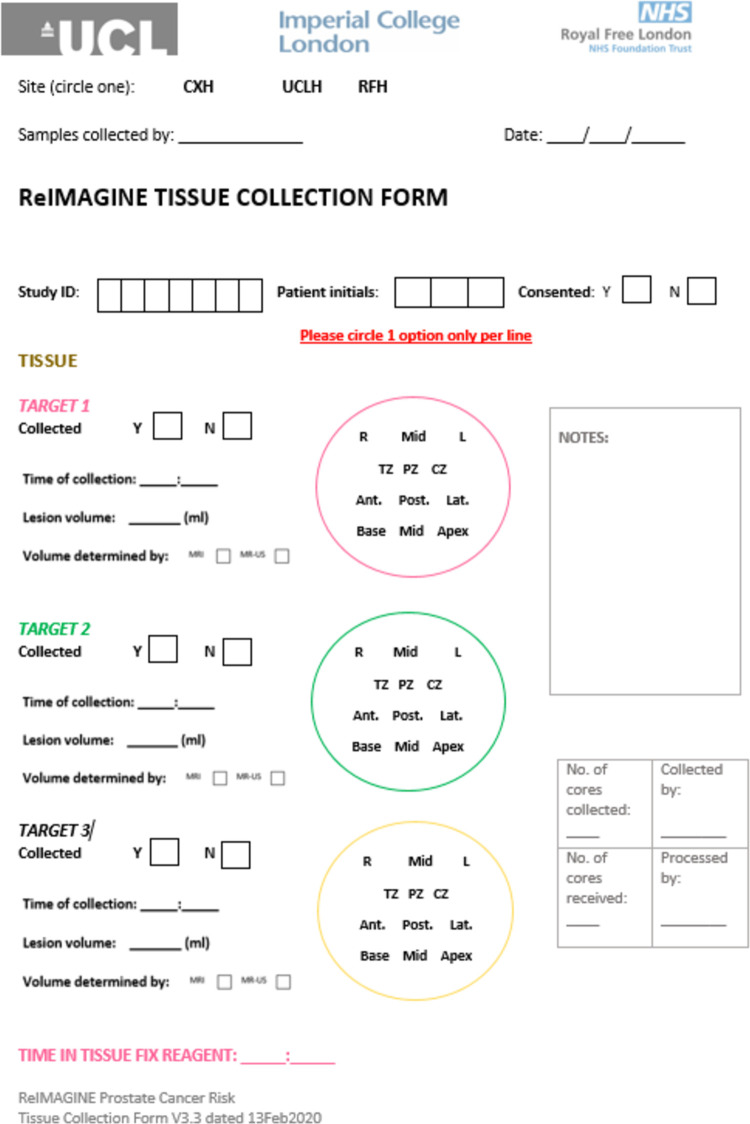
ReIMAGINE prostate cancer risk tissue collection form denoting the location of each donated prostate tissue research core. Consenting patients will donate three research cores of prostate tissue at the time of their standard of care NHS prostate biopsy and the location of each will be recorded on a ReIMAGINE Prostate Cancer Risk tissue collection form. The first research core will be collected from the centre of the lesion with the highest Likert / PIRADSv2.1 score and greatest volume (lesion 1). The second sample will be collected from the centre of lesion 1 unless multiple lesions with the same Likert / PIRADSv2.1 score exist. If multiple lesions with the same Likert / PIRADSv2.1 score exist, the second core should be collected from the next largest lesion with the same MRI score. The third and final research core will be collected from an area of radiologically normal tissue (defined as Likert / PIRADSv2.1 1 or 2).

The location of each prostate core will be recorded within a study CRF (Fig 3) and each core placed within a colour coded pathology cassette before being placed within PAXgene® tissue fixative. Samples will be fixed in PAXgene® Tissue Fix reagent for at least 2–24 hours. Cores are then treated with PAXgene® Tissue Stabalizer concentrate and ethanol for at least 2 hours. All pseudonymised samples will be logged to the FreezerPro database and stored at -80°C following stabaliser treatment.

All biological samples are processed and stored in line with the ReIMAGINE Risk Laboratory Manual. When indicated, samples will be transferred to consortium partners according to study standardised operating procedures and material transfer agreements (MTAs).

### 4.5 Formalin-Fixed Paraffin-Embedded (FFPE) tissue blocks and Haematoxylin and Eosin (H&E) slide collection

In addition to the three prostate tissue cores collected purely for research purposes (Table 4), standard of care diagnostic pathology FFPE tissue blocks and H&E slides will be requested from each participating centre once all diagnostic evaluation is complete. Pseudonymised H&E slides will be scanned using the Hamamatsu Nanozoomer S360 high-throughput digital slide scanner at a resolution of 0.23 μm/pixel. Digitalised, pseudonymised slides will be stored within a dedicated ReIMAGINE database held securely on UCL servers. Digitalised images, after additional de-identification, will be exported via the XNAT platform to the ReIMAGINE Clinical Data Lake (CDL) hosted by Philips in the EU to facilitate data sharing.

Digitalisation will facilitate computer-based assessment of morphological features and central pathology review. ReIMAGINE tissue technicians will also collect tissue slices from standard of care FFPE tissue blocks to provide additional tissue for analysis by consortium partners. All FFPE tissue blocks and H&E slides will then be returned to the relevant NHS pathology department. All processes will be conducted in accordance with study SOPs.

## 5 Outcomes

A lack of consensus regarding the definition of clinically significant prostate cancer remains. Much debate is focused upon the heterogenous cohort of intermediate risk, Gleason 7 disease. Recent studies have applied varied definitions of clinical significance including primary Gleason pattern 4, any grade of cancer core length ≥ 6mm [4] or any core biopsy sample indicating Gleason 3 + 4 disease or greater [11].

By defining the primary outcome of the cross-sectional arm of ReIMAGINE Risk as the presence of any Gleason pattern 7 disease or greater we will gain insight into the baseline characteristics associated with intermediate risk disease which is likely to progress. Such information will permit greater stratification within this heterogenous cohort and further elucidate the definition of clinical significance.

Longitudinal objectives achieved through long-term health data linkage include time to metastasis and / or prostate cancer related death. Longitudinal data will provide meaningful clinical correlation for novel biomarkers and risk models. Primary and secondary outcomes are presented in Table 5. Study outcomes will be published in peer-reviewed journal articles once available.

**Table 5:** Primary and secondary outcomes of the ReIMAGINE prostate cancer risk study.

**Primary outcome: Cross-sectional component**
Presence of clinically significant prostate cancer confirmed on biopsy, defined as any Gleason pattern 7 or greater.
**Primary outcome: Longitudinal component**
Time to metastasis and/or prostate cancer related death.
**Secondary Outcomes**
Time to new prostate cancer in men without cancer at baseline.
Time to prostate cancer specific death and all-cause death in all men.
Time to cancer progression in men identified with prostate cancer at baseline.
*Progression is defined as the need for salvage therapy to the prostate following the primary treatment strategy*, *or*, *need for systemic therapy for prostate cancer treatment following failure of the primary treatment strategy*.

Primary and secondary outcomes of the ReIMAGINE Risk study include both cross-sectional and longitudinal components. This data will inform novel prognostic models for prostate cancer and provide a platform for the discovery and calibration of the full range of prostate cancer diagnostic tests (both commercial and academic).

### 5.1 Mechanistic opportunity

The study provides further opportunity for discovery beyond the predefined primary and secondary outcomes. ReIMAGINE Risk biobanks will present an opportunity for computer-aided diagnostics, machine learning, calibration of commercial biomarkers (blood, urine, tissue and radiomic), the identification of MRI endotype molecular signatures and the stratification of MRI endotypes using clinical, radiomic and molecular inputs.

## 6 Statistical considerations

### 6.1 Sample size calculation

During the PRECISION study, 65% of patients with a PIRADS lesion ≥ 3 were found to have any cancer (Gleason score 3 + 3 or greater) detected on targeted prostate biopsy and 50% of targeted histology from PIRADS ≥ 3 lesions was considered clinically significant (Gleason score 3 + 4 or greater) [11]. Within the population of 1000 recruited participants during ReIMAGINE Risk, a conservative estimate will expect 60% of the cohort to have at least Gleason 3 + 3 disease on targeted histology of which two thirds will be considered clinically significant, defined as any Gleason pattern 7 or greater. Therefore, for the cross-sectional component of the analysis we expect 396 cases of clinically significant cancer and 604 cases of no cancer or clinically insignificant cancer.

Professional consensus is moving away from disease specific death rates towards metastatic progression as the endpoint with the greatest face, criterion and construct validity [12]. Time to metastasis and / or prostate cancer related death defines the primary outcome of the longitudinal component of the study. A cohort of 1000 men with an abnormal MRI should generate an event rate (time to metastases) of 10–20% at 10 years [13]. A conservative view would expect 100 events at 10 years.

### 6.2 Statistical analysis

Statistical analysis will be performed according to pre-specified statistical methodology before the inspection of any outcome data. Logistic regression modelling will be used to analyse the factors associated with presence of clinically significant cancer. Cox regression modelling (or other appropriate survival methods if there is evidence of non-proportional hazards) will be used to analyse the factors associated with time to new cancers, cancer progression, metastasis and death. A list of pre-specified factors will be agreed and these will be systematically analysed using the aforementioned models in order to identify factors which are independently associated with each outcome.

In addition to the simple frequentist analysis approach, a more sophisticated analysis will be conducted. We will first apply the SaddlePoint Signature multivariate Bayesian regression pipeline, which was designed specifically for regression with high-dimensional covariates, in order to generate (i) optimal predictive covariate selections (suppressing false positive associations), (ii) quantitative characteristics of true associations (including covariate interactions, if relevant), (iii) personalised multivariate risk signatures, and (iv) quantifiers of the degree of outcome predictability. This will be done both for the full covariate set and for the individual covariate streams (e.g. imaging, genomic, phenotypic), allowing us to compare the outcome prediction power of different information sources, and their potential synergy. The most recent version of the pipeline (version 2.10.1), uses advanced overfitting correction protocols (based on the replica method) and robust internal validation processes. The risk scores for the separate streams will provide intuitive visualisations of the heterogeneity of the patient cohort. In a second stage we will apply Bayesian multivariate latent class analysis, using the source-specific risk signatures computed in the first stage as meta-covariates, and assess whether and how the cohort should be further stratified into subgroups with distinct associations and/or base hazard rates.

## 7 Ethical considerations

The study abides by the principles of the Declaration of Helsinki and received regulatory approval from the Regional Ethics Committee (London–Stanmore 19/LO/1128). ReIMAGINE Prostate Cancer Risk is published on clinicaltrials.gov (ClinicalTrials.gov identifier: NCT04060589).

## 8 Discussion and limitations

The prostate cancer diagnostic pathway has, until very recently, relied upon PSA-informed systematic biopsy, which is a method prone to considerable sampling error. Such inaccuracy has skewed risk calculators, rendered studies unrepresentative and quite probably misinformed healthcare policy. This represents a significant burden of diagnostic imprecision and accounts for a high burden of over-diagnosis and over-treatment as well as missed diagnoses of clinically important cancers. The use of pre-biopsy mpMRI corrects much of this inaccuracy and MRI positivity is closely correlated to tumour grade and volume—two surrogates of disease significance [4].

The ReIMAGINE consortium will exploit radiomic, molecular and targeted histological data to define novel and measurable image-based disease cohorts. Data from deeply phenotyped MRI cohorts and national healthcare records will provide inputs for progression modelling to more accurately predict disease status and outcomes. Exome and genome analysis of localised prostate and non-small-cell lung cancers suggest an association between intratumoural heterogeneity and poor prognosis [14, 15]. We therefore expect that integrated clinical, molecular (whether fluidic or tissue-based) and imaging analysis will provide more accurate risk stratification than histological assessment–the current gold standard diagnostic test. Molecular profiling of advanced prostate cancers undertaken by other groups will provide a comparator for deeply phenotyped, organ-confined cancer cohorts within ReIMAGINE and add context to our results [16].

ReIMAGINE will also work with industry partners to facilitate the discovery, calibration and validation of fluidic biomarkers in blood and urine. Genome and exome analysis of plasma-extracted circulating tumour DNA has shown promise during the prediction of disease outcomes in advanced prostate cancer as well as the prediction of local disease recurrence in other cancer types [17, 18]. Plasma-extracted cell-free DNA is also used to guide the treatment of advanced non-small cell lung cancers (NSCLC) [19], whilst commercially available urinary exosome and methylome tests show great potential for predicting clinically significant prostate cancer more accurately than PSA [20, 21].

### 8.1 Limitations

The ReIMAGINE Risk protocol has some potential limitations. First, novel biomarker tests will be calibrated and validated against a population in whom a standard of care prostate biopsy has been deemed necessary following assessment with mpMRI. Biomarkers will therefore not be calibrated to patients who have not had MR imaging or in whom prostate mpMR imaging is unremarkable, however this could form part of further work. A prostate tissue sample from an area of Likert / PIRADSv2.1 score less than or equal to 2 will be collected from each participant to mitigate against the absence of a normal MRI cohort and act as a control. We also anticipate around one third of participants will have altogether benign prostate sampling, and will therefore form a further comparator group, albeit not representative of radiologically normal prostates as there is a requirement for an mpMRI Likert / PIRADSv2.1 score of 3 or greater to enter the study.

Second, many patients referred for secondary care assessment and therefore captured within the study population are referred because of a raised PSA level. Further work may be required to assess the applicability of findings to a cohort of men with a normal PSA. A number of patients within ReIMAGINE Risk will be recruited following participation within the ReIMAGINE screening study. ReIMAGINE screening has been designed to assess the feasibility of bi-parametric prostate MRI as a screening tool for prostate cancer. Patients may screen positive on the basis of either, or both, MRI positivity and raised PSA density. This cohort may broaden the spectrum of patients recruited to ReIMAGINE Risk to include a greater proportion of participants with a normal PSA.

Third, within participating centres, prostate cancer risk assessment and management decisions are already heavily directed by mpMRI findings. Longitudinal study outcomes may, therefore, fail to accurately reflect outcomes conferred by traditional risk stratification models and under-represent the need for disease re-stratification with contemporaneous MRI and targeted histological inputs. However, ReIMAGINE Risk represents the first attempt to redefine risk calculators during a new era of prostate cancer precision diagnostics.

## 9 Conclusion

As we transition to mpMRI-led prostate cancer diagnosis it is imperative that we redefine existing risk stratification models. Current risk models continue to inform practice but are based upon historic tissue archives and clinical cohorts in which TRUS biopsy was the baseline diagnostic test; they therefore cannot readily be applied to patients diagnosed by an mpMRI-targeted approach. ReIMAGINE Risk will deeply phenotype mpMRI disease cohorts and collect long-term oncological outcomes from national healthcare records. Study data will provide image-based inputs for progression modelling and capture the full range of novel prostate cancer diagnostics.

## Supporting information

S1 Checklist SPIRIT 2013 checklist: Recommended items to address in a clinical trial protocol and related documents*. (DOC)

S1 File Appendix I: mpMRI data management. (PDF)

S2 File Appendix II: Blood sample processing. (PDF)

S3 File Appendix III: Material and sample storage. (PDF)

S4 File Appendix IV: Discontinuation/withdrawal of participants. (PDF)

S5 File Appendix V: ReIMAGINE consortium structure, governance and work strands. (PDF)

S6 File Appendix VI: ReIMAGINE consortium partners (Academic and Commercial) at the time of publication. (PDF)

S7 File Appendix VII: Data management model. (PDF)

S8 File Appendix VIII: Acknowledgments. The ReIMAGINE risk study group.
(DOCX)

S9 File (DOCX)

## Abbreviations

eCRF: Electronic Case Report Form
EU: European Union
FFPE: formalin-fixed paraffin-embedded
H&E: haematoxylin and eosin
HTA: Human Tissue Authority
MRI: magnetic resonance imaging
MTA: material transfer agreement
mpMRI: multiparametric magnetic resonance imaging
NHS: National Health Service
PCa: prostate cancer
PSA: prostate specific antigen
REC: Research Ethics Committee
SOP: Standard Operating Procedure
TRUS: Transrectal Ultrasound
UCL: University College London
