# Supplementary material for: The ReIMAGINE prostate cancer risk study protocol: A prospective cohort study in men with a suspicion of prostate cancer who are referred onto an MRI-based diagnostic pathway with donation of tissue, blood and urine for biomarker analyses
Source: PLoS One. 2022 Feb 24;17(2):e0259672. doi: 10.1371/journal.pone.0259672 (PMC8870538; doi:10.1371/journal.pone.0259672)
Supplement: S1 File — (PDF) [file pone.0259672.s002.pdf]

Appendix I: mpMRI data management

Pseudonymised DICOM (Digital Imaging and Communications in Medicine) images from each participant's pre-biopsy standard of care mpMRI will be uploaded to the Cancer Research U.K. (CRUK) funded National Cancer Imaging Translational Accelerator (NCITA) image repository, hosted by UCL. A copy of these images, adhering to additional de-identification parameters, will be transferred via the XNAT platform to the ReIMAGINE Clinical Data Lake hosted by Philips in the European Union. Both platforms have strict safeguards against unauthorised access with established security procedures. Images stored within the data warehouse may be accessed by consortium partners and shared with the wider research community (Appendix VII). Archiving, quality control and triaging of studies will be conducted prior to quantitative processing by an experienced radiologist and archive manager.
