# Supplementary material for: The ReIMAGINE prostate cancer risk study protocol: A prospective cohort study in men with a suspicion of prostate cancer who are referred onto an MRI-based diagnostic pathway with donation of tissue, blood and urine for biomarker analyses
Source: PLoS One. 2022 Feb 24;17(2):e0259672. doi: 10.1371/journal.pone.0259672 (PMC8870538; doi:10.1371/journal.pone.0259672)
Supplement: S2 File — (PDF) [file pone.0259672.s003.pdf]

S2 File: Appendix II: Blood sample processing

EDTA processing

Samples collected in EDTA vials will be centrifuged in a two-spin process at 4°C: 1600g for 15 minutes, followed by 3000g for 10 minutes (plasma only).

SST processing

Blood will be allowed to clot at room temperature for at least 30 minutes after sample collection, refrigerated and centrifuged at 1600g for 10 minutes at 4°C (with a break) no later than 4 hours after sample collection.

Strek™ processing

Strek™ vial samples will be centrifuged in a two-spin process at room temperature: 1600g for 15 minutes, followed by 3000g for 10 minutes.

PAXgene® Blood RNA

Blood samples will be stored in PAXgene® Blood RNA vials for at least two hours after collection and stored in the original collection tubes at -20°C for 24 – 48 hours followed by long-term storage at -80°C.
