# Supplementary material for: The ReIMAGINE prostate cancer risk study protocol: A prospective cohort study in men with a suspicion of prostate cancer who are referred onto an MRI-based diagnostic pathway with donation of tissue, blood and urine for biomarker analyses
Source: PLoS One. 2022 Feb 24;17(2):e0259672. doi: 10.1371/journal.pone.0259672 (PMC8870538; doi:10.1371/journal.pone.0259672)
Supplement: S3 File — (PDF) [file pone.0259672.s004.pdf]

S3 File: Appendix III: Material and sample storage

Samples will be processed, stored and disposed of in accordance with all applicable ethical, legal and regulatory requirements, including the Human Tissue Act 2004 [22] and any amendments thereafter. Biological samples will be stored under the Human Tissue Authority (HTA) licence 12055 and shipped periodically to academic and commercial partners in the EU, North America and Australia for analyses.

Academic and commercial partners will require a material (and data) transfer agreement (MTA) before shipment of any biological samples is permitted from the central UCL laboratory, or access/electronic transfer of data is facilitated. Partners requesting only data (radiomics, imaging or clinical) will require a data transfer agreement (DTA) prior to any transfer. Access to samples by Consortium partners is subject to review by the ReIMAGINE biological research group (Appendix V) who will maintain scientific rigour and prioritise material transfer accordingly. Biological samples will be disposed of, or transferred to a licenced tissue bank, in accordance with the Human Tissue Act 2004[22] and any amendments thereafter once ethical approval for the study has expired.
