# Supplementary material for: The ReIMAGINE prostate cancer risk study protocol: A prospective cohort study in men with a suspicion of prostate cancer who are referred onto an MRI-based diagnostic pathway with donation of tissue, blood and urine for biomarker analyses
Source: PLoS One. 2022 Feb 24;17(2):e0259672. doi: 10.1371/journal.pone.0259672 (PMC8870538; doi:10.1371/journal.pone.0259672)
Supplement: S4 File — (PDF) [file pone.0259672.s005.pdf]

S4: Appendix IV: Discontinuation/withdrawal of participants

Participants can be withdrawn at any time if involvement in the study is no longer considered to be in their best interests, consent is withdrawn or compliance with protocol requirements is not possible. The reason for withdrawal will be documented in both the eCRF and medical record. Any data collected prior to withdrawal will be kept in accordance with the Data Protection Act (2018) and General Data Protection Regulations (GDPR). Donation of less than 10ml of blood (1 x 10ml EDTA) or urine or less than 1 prostate tissue core is considered a protocol deviation.
