# Supplementary figures and images for: The ReIMAGINE prostate cancer risk study protocol: A prospective cohort study in men with a suspicion of prostate cancer who are referred onto an MRI-based diagnostic pathway with donation of tissue, blood and urine for biomarker analyses
Source: PLoS One. 2022 Feb 24;17(2):e0259672. doi: 10.1371/journal.pone.0259672 (PMC8870538; doi:10.1371/journal.pone.0259672)

## S5 File: Appendix V: ReIMAGINE Consortium structure, governance and work strands

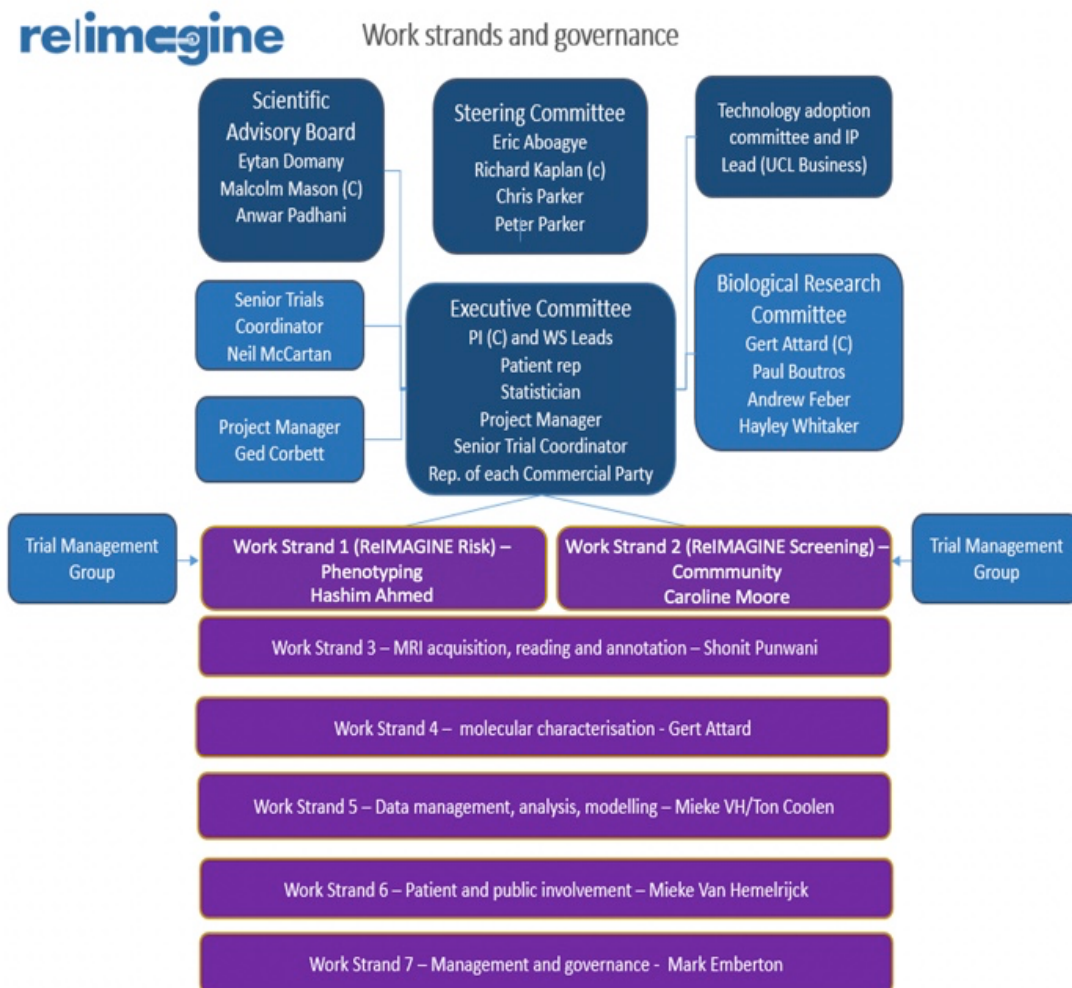

Supplement: S5 File — (PDF) [file pone.0259672.s006.pdf]
