# Supplementary material for: The ReIMAGINE prostate cancer risk study protocol: A prospective cohort study in men with a suspicion of prostate cancer who are referred onto an MRI-based diagnostic pathway with donation of tissue, blood and urine for biomarker analyses
Source: PLoS One. 2022 Feb 24;17(2):e0259672. doi: 10.1371/journal.pone.0259672 (PMC8870538; doi:10.1371/journal.pone.0259672)
Supplement: S6 File — (PDF) [file pone.0259672.s007.pdf]

S6 File: Appendix VI: ReIMAGINE Consortium Partners (Academic and Commercial) at the time of publication

(1) University College London

(2) King's College London

(3) Imperial College London

(4) London Institute for Mathematical Sciences

(5) Chronix Biomedical Inc.

(6) Cortechs.at

(7) Exosome Diagnostics

(8) Decipher Biosciences, Inc.

(9) Ibex

(10) iCAD Inc.

(11) Image Analysis Limited

(12) Lucida Medical

(13) Maxwell Plus

(14) MDx Health

(15) MIM Software Inc.

(16) Minomic International Limited

(17) Philips Electronics Nederland BV

(18) Proteomedix

(19) Watson Medical
