# Supplementary material for: The ReIMAGINE prostate cancer risk study protocol: A prospective cohort study in men with a suspicion of prostate cancer who are referred onto an MRI-based diagnostic pathway with donation of tissue, blood and urine for biomarker analyses
Source: PLoS One. 2022 Feb 24;17(2):e0259672. doi: 10.1371/journal.pone.0259672 (PMC8870538; doi:10.1371/journal.pone.0259672)
Supplement: S8 File — The ReIMAGINE risk study group. (DOCX) [file pone.0259672.s009.docx]

**S8 File: Appendix VIII: Acknowledgements**

The ReIMAGINE Risk Study Group

| **Trial Sponsor:** | University College London (UCL) |
| --- | --- |
| **Trial Coordination:** | UCL Urology, Research Department of Targeted Intervention, Division of Surgery and Interventional Science |
| **Funders:** | The Medical Research Council, U.K. (MRC), grant number MR/R014043/1 and Cancer Research U.K. (CRUK) |
| **Group Lead Author:** | Teresa Marsden (teresa.marsden@ucl.ac.uk) |

| **Name** | **Institution** | **Role** |
| --- | --- | --- |
| Professor Mark Emberton | UCL, UCLH | WS lead and/or co-applicant |
| Professor Hashim U Ahmed | ICL, CXH | WS lead and/or co-applicant |
| Professor Caroline Moore | UCL, UCLH | WS lead and/or co-applicant |
| Professor Mieke Van Hemelrijck | KCL | WS lead and/or co-applicant |
| Professor Shonit Punwani | UCL, UCLH | WS lead and/or co-applicant |
| Dr Manuel Rodriguez-Justo | UCLH | WS lead and/or co-applicant |
| Dr Gerhardt Attard | UCL | WS lead and/or co-applicant |
| Professor Louise Brown | UCL MRC | Statistician |
| Ms Elena Frangou | UCL MRC | Statistician |
| Ms Chris Brew-Graves | UCL, NCITA | Co- applicant |
| Professor Ton Coolen | Radboud University | WS lead and/or co-applicant |
| Mr Steve Tuck | Patient representative | WS lead and/or co-applicant |
| Dr Aida Santa Olalla | KCL | Data Management |
| Ms Charlotte Moss | KCL | Data Management |
| Ms Saran Green | KCL | PPI |
| Mr Neil McCartan  Mrs Rosie Clow | UCL, UCLH, ICL  UCL | Clinical Project Manager  Clinical Trial Manager |
| Mr Ged Corbett | UCL TRO | Project Manager |
| Mrs Anna Wingate  Dr Teresa Marsden  Ms Joanna Hadley  Ms Fatima Akbar  Ms Hina Pervez  Ms Suparna Thakali  Ms Ashling Henderson  Ms Dizem Tekin  Dr Giorgio Brembilla  Dr Francesco Giganti  Dr Tom Syer  Mr Joey Clement  Dr Harbit Sidhu | UCL, UCLH | Site staff |
| Ms Elizabeth Isaac  Ms Teresita Beeston  Ms Katerina Soteriou | UCLH | Site staff |
| Ms Francesca Rawlins  Ms Pirruntha Sivaharan  Ms Kinnari Naik  Ms Savahnna Wolfe  Dr Henry Tam  Ms Heather Bholastewart  Dr Sarp Keskin  Ms Mariana Bertoncelli  Mr William Maynard | ICL, CXH | Site staff |
| Professor Charlotte Bevan  Dr Paul Boutros  Dr Andrew Feber  Dr Hayley Whitaker  Dr Alex Freeman | ICL  UCLA  ICR  UCL  UCLH | BRC |
| Professor Caroline Dive  Professor Eytan Domany  Professor Malcolm Mason (C)  Professor Anwar Padhani | CRUK / University of Manchester  Weizmann Institute of Science  Cardiff University  ICR | SAB |
| Professor Eric Aboagye  Professor Richard Kaplan (c)  Professor Chris Parker  Professor Peter Parker | ICL  UCL MRC  Royal Marsden & ICR  KCL | SC |
| Mr Lee Berney  Mr Andrew Prrugia | NOCLOR | NOCLOR |
| Jayshireen Singh | CRN | CRN |
