# Supplementary material for: The ReIMAGINE prostate cancer risk study protocol: A prospective cohort study in men with a suspicion of prostate cancer who are referred onto an MRI-based diagnostic pathway with donation of tissue, blood and urine for biomarker analyses
Source: PLoS One. 2022 Feb 24;17(2):e0259672. doi: 10.1371/journal.pone.0259672 (PMC8870538; doi:10.1371/journal.pone.0259672)
Supplement: S9 File — (DOCX) [file pone.0259672.s010.docx]

**ReIMAGINE:**

**A prospective cohort study in men with a suspicion of prostate cancer who are referred onto an MRI-based diagnostic pathway with donation of tissue, blood and urine for biomarker analyses**

**ReIMAGINE Prostate Cancer Risk**

**Co-Chief Investigators:**

Professor Hashim Ahmed

Professor Mark Emberton

**Supported by:**

The Medical Research Council and CRUK

**Sponsored by:**

University College London (UCL)

**Protocol version number and date:**

Version 2.1, dated 12Nov2019

**R&D / Sponsor Reference Number(s):**

123973

**Study Registration Number:**

NCT04060589

**PROTOCOL VERSIONS**

| **Versions No** | **Version Date** | **Protocol updated & finalised by;** | **Appendix No detail the reason(s) for the protocol update** |
| --- | --- | --- | --- |
| 1.1 | 17^th^ July 2019 | **Updated by**: Neil McCartan  **Finalised by:** Hashim Ahmed | Updated in line with REC feedback |
| 2.0 | 5^th^ August 2019 | **Updated by**: Neil McCartan  **Finalised by:** Hashim Ahmed | Updated in line with REC feedback |
| 2.1 | 12^th^ Nov 2019 | **Updated by**: Neil McCartan  **Finalised by:** Hashim Ahmed | The protocol has been amended to allow men to participate if they have a PSA value 20 ng/ml or less with the value recorded <12 months before study entry. |
|  |  |  |  |

**DECLARATIONS**

The undersigned confirm that the following protocol has been agreed and accepted and that the investigator agrees to conduct the study in compliance with the approved protocol and will adhere to the Research Governance Framework 2005 (as amended thereafter), the General Data Protection Guidance (GDPR), the Trust Data & Information policy, Sponsor and other relevant SOPs and applicable Trust policies and legal frameworks.

I (investigator) also confirm that an honest accurate and transparent account of the study will be given; and that any deviations from the study as planned in this protocol will be explained and reported accordingly.

**Chief Investigator:**

**Signature Date** 12^th^ November 2019

**Print Name (in full):** Professor Hashim Uddin Ahmed

**Position:** Professor of Urology, Chair of Urology and Consultant Urological Surgeon

**On behalf of the Study Sponsor:**

**Signature: Date:** 13th November 2019

**Print Name (in full):** Pushpsen Joshi

**Position:** Research Governance Manager

**STUDY SUMMARY**

| **Identifiers** | | |
| --- | --- | --- |
| IRAS Number | 251166 | |
| REC Reference No | 19\LO\1128 | |
| Sponsor Reference No | 123973 | |
| Other research reference number(s) (if applicable) | CPMS: 42576 | |
| Full (Scientific) title | A prospective cohort study in men with a suspicion of prostate cancer who are referred onto an MRI-based diagnostic pathway with donation of tissue, blood and urine for biomarker analyses | |
| Health condition(s) or problem(s) studied | Prostate cancer | |
| Study Type | Single arm multi-site prospective longitudinal cohort study with blood, urine and tissue donation | |
| Target sample size | Men with PIRADS/LIKERT MRI lesions of score 3, 4 or 5 undergoing a targeted and systematic biopsy | |
| **STUDY TIMELINES** | | |
| Study Duration/length | **First patient recruited:** 1^st^ September 2019  **Last patient recruited:** 31^st^ May 2021  **Follow-up:** Indirect data collection via healthcare data linkage 31^st^ May 2024 | |
| Expected Start Date | 1^st^ September 2019 | |
| End of Study definition and **anticipated date** | **Cross Sectional component**   - **LPLV:** 31^st^ May 2021 - **Database Lock:** 31^st^ May 2022   **Longitudinal component**   - **3 years after LPLV:** 31^st^ May 2024 | |
| **FUNDING & Other** | | |
| Funding | Medical Research Council (MRC) and Cancer Research UK (CRUK) | |
| Other support | Commercial organisations that sign up to the consortium agreement to become ReIMAGINE partners will be providing *in-kind* contributions. | |
| **STORAGE of SAMPLES** | | |
| Human tissue samples | **Biological samples will be transferred to and processed at:**  The laboratories of Professor Gert Attard, at UCL Cancer Institute, Paul O'Gorman Building, 72 Huntley Street, London, WC1E 6DD  Aliquots will be sent to partners as per the ReIMAGINE Consortium agreement. | |
| Data collected / Storage | All identifiable data will be held on secure servers hosted and maintained by University College London (UCL).  Pseudonymised data, including imaging and histology data, will be held by UCL and transferred to central secure servers held in the EU and managed by Philips.  Pseudonymised MRI DICOM images and related annotations will be uploaded to the CRUK funded National Cancer Imaging Translational Accelerator (NCITA) image repository before a copy is shared with Philips via the XNAT platform. | |
| **KEY STUDY CONTACTS** |  | |
| Chief Investigator  **Professor Hashim Ahmed**  University College London | | Co-Chief Investigator  **Professor Mark Emberton**  University College London |

**KEY ROLES AND RESPONSIBILITIES**

**SPONSOR:** The sponsor is responsible for ensuring that, prior to commencement of a study, the necessary arrangements are in place for the research team to access resources and support the delivery of the research as proposed. The Sponsor is equally responsible for allocating responsibilities for the management, monitoring and reporting of the research. Additionally, the Sponsor must be satisfied that there is agreement on appropriate arrangements to record, report and review significant developments as the research proceeds, and approve any modifications to the study design.

**FUNDER:** The funder is the entity that will provide the funds (financial support) for the conduction of the study. Funders are expected to provide assistance to any enquiry, audit or investigation related to the funded work.

**CHIEF INVESTIGATOR (CI):** The person who takes overall responsibility for the design, conduct and reporting of a study. If the study involves researchers at more than one site, the CI takes on the primary responsibility whether or not he/she is an investigator at any particular site.

The role of the CI is to complete and to ensure that all relevant regulatory approvals are in place before the study begins. They are also responsible for ensuring arrangements are in place for good study conduct, robust monitoring and reporting, including prompt reporting of incidents. Such processes include the provision of adequate training for study staff to conduct the study as per the protocol and relevant standards.

The Chief Investigator is responsible for the submission of annual reports as required. The Chief Investigator will notify the Research Ethics Committee (REC) of the end of the study, including any reasons for the premature termination. Within one year after the end of study, the Chief Investigator will submit a final report with the results, including details of any publications/abstracts, to the REC.

**PRINCIPLE INVESTIGATOR (PI):** Individually or as leader of the researchers at a site; ensuring that the study is conducted as per the approved study protocol, and reporting/notifying the relevant parties – this includes the CI of any breaches or incidents related to the study.

**LIST OF ABBREVIATIONS**

AE Adverse Event

CI Chief Investigator

eCRF Electronic Case Report Form

CRO Contract Research Organisation

DMC Data Monitoring Committee

EU European Union

GAfREC Governance Arrangements for NHS Research Ethics

GCP Good Clinical Practice

HTA Human Tissue Authority

ICF Informed Consent Form

IDMC Independent Data Monitoring Committee

IMP Investigational Medicinal Product

ISF Investigator Site File

ISRCTN International Standard Randomised Controlled Trial Number

LPLV Last patient last visit

MRI Magnetic Resonance Image

mpMRI Multi-parametric MRI

NHS R&D National Health Service Research & Development

PI Principal Investigator

PIS Participant Information Sheet

QA Quality Assurance

QC Quality Control

RCT Randomised Controlled Trial

REC Research Ethics Committee

SAE Serious Adverse Event

SC Steering Committee

SDV Source Document Verification

SOP Standard Operating Procedure

TMG Trial Management Group

TRUS Transrectal Ultrasound

CONTENTS

[1 INTRODUCTION 9](#_Toc14683873)

[2 BACKGROUND AND RATIONALE 11](#_Toc14683874)

[3 OBJECTIVES 13](#_Toc14683875)

[3.1 Primary Outcome 13](#_Toc14683876)

[3.1.1 Cross sectional component primary outcome 13](#_Toc14683877)

[3.1.2 Longitudinal Component primary outcome 13](#_Toc14683878)

[3.2 Secondary Outcomes 13](#_Toc14683879)

[4 STUDY DESIGN 14](#_Toc14683880)

[5 STUDY SCHEDULE 14](#_Toc14683881)

[5.1 Screening and Registration 14](#_Toc14683882)

[5.2 Discontinuation/withdrawal of participants 20](#_Toc14683883)

[5.3 Definition of End of Study 20](#_Toc14683884)

[6 CONSENT 21](#_Toc14683885)

[7 ELIGIBILITY CRITERIA 22](#_Toc14683886)

[7.1 Inclusion Criteria 22](#_Toc14683887)

[7.2 Exclusion Criteria 23](#_Toc14683888)

[8 RECRUITMENT 23](#_Toc14683889)

[9 STATISTICAL METHODS 23](#_Toc14683890)

[10 PATIENT AND PUBLIC INVOLVEMENT (PPI) 24](#_Toc14683891)

[11 FUNDING AND SUPPLY OF EQUIPMENT 25](#_Toc14683892)

[12 DATA HANDLING AND MANAGEMENT 25](#_Toc14683893)

[13 MATERIAL/SAMPLE STORAGE 27](#_Toc14683894)

[13.1 Blood Sample Collection 28](#_Toc14683895)

[13.2 Urine sample collection 28](#_Toc14683896)

[13.3 Prostate tissue Collection 29](#_Toc14683897)

[13.4 Paraffin and H&E slide collection 29](#_Toc14683898)

[13.5 Imaging databank 30](#_Toc14683899)

[14 PEER AND REGULATORY REVIEW 30](#_Toc14683900)

[15 ASSESMENT AND MANAGEMENT OF RISK 30](#_Toc14683901)

[16 RECORDING AND REPORTING OF EVENTS AND INCIDENTS 31](#_Toc14683902)

[16.1 Recording adverse events 31](#_Toc14683903)

[16.2 Procedures for recording and reporting Serious Adverse Events 31](#_Toc14683904)

[17 PROTOCOL DEVIATIONS AND NOTIFICATION OF PROTOCOL VIOLATIONS 32](#_Toc14683905)

[18 TRUST INCIDENTS AND NEAR MISSES 32](#_Toc14683906)

[19 MONITORING AND AUDITING 32](#_Toc14683907)

[20 TRAINING 33](#_Toc14683908)

[21 INDEMNITY ARRANGEMENTS 33](#_Toc14683909)

[22 STUDY GOVERNANCE 33](#_Toc14683910)

[22.1 Responsibilities 33](#_Toc14683915)

[22.2 Operational Structure 33](#_Toc14683916)

[22.3 Oversight/ Study Monitoring Groups 34](#_Toc14683917)

[23 ARCHIVING 34](#_Toc14683918)

[24 PUBLICATION AND DISSEMINATION POLICY 35](#_Toc14683919)

[25 REFERENCES 36](#_Toc14683920)

[26 APPENDIX I: Oversight/Study monitoring Group Membership 37](#_Toc14683921)

[28 APPENDIX II: Schedule of assessments 39](#_Toc14683922)

[29 APPENDIX III: ReIMAGINE Consortium partners 40](#_Toc14683923)

# INTRODUCTION

Prostate cancer diagnosis has undergone considerable change over the last decade with an increasing uptake of multi-parametric MRI (mpMRI) prior to prostate biopsy. In the past, most men underwent a transrectal ultrasound guided (TRUS) biopsy without knowledge of whether a man had cancer or not, and if there was a cancer, blind to its location. Currently, UK practice is approaching a point where mpMRI is likely to become part of the standard-of-care with the UK’s National Institute for Health and Care Excellence (NICE) issuing guidance recommending that an mpMRI be offered to all men prior to a prostate biopsy ([www.nice.org.uk/guidance/indevelopment/gid-ng10057](http://www.nice.org.uk/guidance/indevelopment/gid-ng10057)).

This modification to the prostate cancer diagnostic pathway has resulted in significant consequences to men at risk. These are likely to comprise the following based on the evidence accumulated in high quality prospective studies: fewer men biopsied overall; fewer needle deployments; less over-diagnosis; fewer missed clinically important cancers; and less overall harm to the patients (Ahmed H *et al.* Lancet 2017; Kasivisvanathan V *et al.* NEJM 2018). These benefits arise from the ability of mpMRI to locate clinically significant prostate cancer as mpMRI positivity is positively associated with both tumour grade and volume – the two prostate cancer attributes most associated with risk.

The result is a process of risk stratification that is about 100% better (Ahmed H *et al.* Lancet 2017). than that which preceded the mpMRI based approach and results in a concordance with verified pathology on whole-gland step section (following surgical removal) of around 90% (Baco E *et al.* Eur Urol 2015).

This new level of precision creates problems as all the risk models currently in use for prostate cancer risk estimation are based on data derived from a TRUS biopsy pathway. Because the methods prior to mpMRI used a combination of PSA and transrectal ultrasound (TRUS) guided biopsy men, invariably, had their risk under-estimated in a number of ways. First, they were told they were clear when they were not. Second, few men with anterior disease were ever diagnosed. Third, the Gleason grade was under-estimated in 30-50% of cases. The result of these risk-stratification errors – perhaps the greatest in all of the cancer field – was that men had either no treatment (when they needed it), or delayed treatment (when it should have been given quicker) or inappropriate treatment (when it was not needed at all due to an unimportant indolent cancer diagnosis).

The other consequence relates to our understanding of prostate cancer – and is much less well appreciated. This is the fact that the spectrum of cancer that we have been diagnosing for the last 50 years is a function of the performance of the tests we have used historically and is therefore considerably skewed against the true spectrum of disease that exists.

There exist known and unknown errors that will result from applying old risk-stratification methods to the new outputs of an image-based diagnostic pathway. The main error that results is that risk will be over-estimated (when cancer is present) for most of the men that are diagnosed if we superimpose the histological profile of tissue acquired as a result of an MRI-targeted biopsy as opposed to a TRUS guided biopsy in the same man. Typically, the TRUS-biopsy derived tissue will have fewer cores that are positive, reduced maximum cancer core lengths and a lower overall Gleason attribution.

As there is no obvious method to adjust for this error the general view is that new and better risk-stratification and prediction models will need to be created using inputs that are derived from men undergoing a contemporary assessment. It was to address this particular challenge that the ReIMAGINE Consortium was conceived of and created.

**ReIMAGINE Prostate Cancer Risk** constitutes the principal component of ReIMAGINE and was designed to risk-stratify men as accurately as is currently possible within a contemporary MR-imaging-based diagnostic pathway. It was designed to create an opportunity for the many commercial entities that exist that have developed biomarkers to assist in making the risk stratification of these men more precise. Most, if not all, of these biomarkers were developed to correct the errors associated with TRUS guided biopsy. They now would benefit from being re-calibrated to the new image-based pathway. ReIMAGINE will create a platform designed to assist in this task – both at cross sectional risk-stratification (precise diagnosis) but also down-stream prediction of key events such as clinical progression, time to metastases and death. Further, ReIMAGINE will permit the discovery and validation of novel clinical, fluidic, histological and imaging biomarkers, alone or in combination, by the biobanking and data banking of patient derived samples.

**Figure 1: ReIMAGINE Prostate Cancer Risk patient flow**


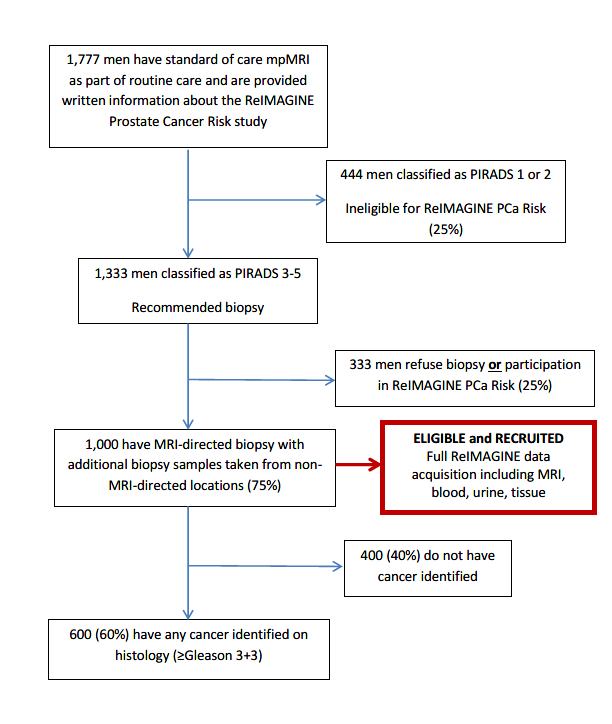


# BACKGROUND AND RATIONALE

For the last 50 years PSA-biopsy risk-stratification has been applied to men at risk of prostate cancer, which is now known to be unfit for purpose. PSA-biopsy risk-stratification results in the greatest burden in all oncology of over-diagnosis, over-treatment, and of missed cancers that are of clinical importance. This systematic error has biased tissue archives, skewed risk calculators, and rendered studies un-representative and undermined policy-making. MRI has been shown to provide almost complete correction for each error associated with the traditional PSA-biopsy approach by identifying a distinct stratum from the wider population at risk that is closely correlated to the two strongest progression predictors - grade and volume. Encouraged by the patient/user groups within the ReIMAGINE consortium, the aim has been to create a novel, image based, measurable-disease endotype and to use it to risk stratify men with prostate cancer and, as a result, improve the ability to derive prognostic information and allocate men to the most appropriate and effective therapies. This will be achieved by creating unique cohorts, each defined by the presence of a specific MRI-derived endotype that will provide clinical, molecular and radiological inputs into a progression model that will be specified to predict disease status, radiological, clinical and metastatic progression. During this process, insight into the key mechanistic events that drive progression will be available for the first time. Machine learning algorithms from the radiology-pathology annotated training data sets will inform computer-assisted diagnosis. There will be a number of commercial partners (that have created biomarkers to risk-stratify, predict and prognosticate) who will generate further their work as a result of the opportunity that ReIMAGINE provides to refine their performance by means of enhanced precision and, in addition, create new risk models that integrate MRI-derived radiomics with their improved proprietary offerings.

ReIMAGINE Prostate Cancer Risk seeks to exploit a novel and improved diagnostic pathway for men at risk of prostate cancer. This pathway is in its most advanced state of implementation in London (UK), largely as a result of its early implementation and on the successful delivery of the PROMIS study - level-1 evidence showing the superiority of MRI over traditional diagnostic measures. For this reason the ReIMAGINE consortium has chosen to base this study in hospital groups where prostate MRI is in an advanced state and offered as standard of care within an NHS setting. Participants will comprise NHS patients who have undergone a high-quality prostate MRI as part of routine NHS care in preparation for a decision on whether or not to proceed to biopsy. In partner hospitals - but not necessarily standard elsewhere - men with abnormalities in their prostates conforming to Likert/PIRADS 3-5 are offered targeted biopsies. It is at this point that men would be approached and informed about the study and their consent sought. As some sites run prostate cancer “one-stop” clinics the protocol will allow the option of explaining and discussing consent over the telephone. The study therefore is not changing the standard of care - merely exploiting it to explore the opportunity to follow these men a little more closely and seek permission to obtain tissue (from the biopsy), blood and urine so that synergies between the imaging phenotype and the manifestation of molecular changes that appear in the tissue and fluid samples can be explored. Once this process is complete, men return to standard of care, but their consent is sought for healthcare data linkage. ReIMAGINE Prostate Cancer Risk is seeking to deeply phenotype 1,688 men in this way in the least disruptive and most cost-efficient way possible.

One other huge opportunity is to use the ReIMAGINE platform to define what it is we mean by the term ‘clinically important’ or ‘clinically significant’ prostate cancer. There is no agreed threshold that makes the literature difficult, if not impossible, to interpret as each study group is seeking to predict a different disease entity. The current consensus is that the presence of any Gleason pattern 4 constitutes clinical significance. This was the definition we used in PRECISION (Kasivisvanathan V *et al.* NEJM 2018). This is almost certainly too low a threshold given the recent publication of the 29-year follow-up of the SPCG-4 study (Bill-Axelson A *et al.* NEJM 2018). This showed that death from prostate cancer was not associated with Gleason 3 plus 4 disease. Risk came from higher grade (Gleason 4 plus 3 or more) and/or extra-capsular extension. In PROMIS any Gleason 4+3 or greater or a cancer core length of greater than 5mm of any grade was used. This is a disease endo-type that is invariably captured by mpMRI (Ahmed et al, Lancet, 2018). Because we are following the ReIMAGINE cohort to ultimate death (with appropriately secured funding) we should be in a good position to define the baseline characteristics that are associated with both a prostate cancer-related death and the important clinical events that tend to precede it.

ReIMAGINE Prostate Cancer Risk will create the first set of cohorts of men with localized prostate cancer in which truly representative tissue will be procured. The result is an opportunity to phenotype prostate cancer in a manner that has not been possible to date. The discernible sub-groups that emerge should, as consequence, be free of the prognostic imprecision that has hampered our attempts at both risk assessment and appropriate treatment allocation.

We hypothesise that tumour molecular complexity and intra-tumour heterogeneity (ITH) integrated with imaging will improve risk stratification over and above the current best predictor– histological grade. Studies have shown that ITH positively associated with poor prognosis in prostate and lung cancer (Fraser N, Nature 2017; Jamal-Hanjani M, NEJM 2017). Ongoing efforts to extensively characterise the genome/exome, transcriptome and methylome of localized and advanced prostate cancer provide a resource of molecular data that we vertically integrate with the profiling of more advanced disease (STRATOSPHERE).

Our industry partners are developing algorithms to detect at high-resolution tumour DNA in plasma that will be evaluated for associations with risk of progression. By integrating with matched tumour multi-region sequencing data and complementing with high-coverage targeted NGS performed separately on the extracted plasma DNA, we aim to identify independent predictors of progression detected in circulation prior to biopsy and track sub-clonal relapse in follow-up samples.

This protocol has been informed, developed and approved in collaboration with patient and public involvement initiatives occurring in parallel with preparation of this submission. Additional experience is based on investigators’ experience in the PROMIS trial which successfully recruited on time and led to a change in pathway, has been incorporated into this protocol (Ahmed H *et al.* Lancet 2017). PRECISION, an international, multi-centre randomised study that compared an mpMRI based pathway with the standard of care (TRUS-Biopsy) showed that the MRI-based pathway was superior in terms of the proportion of men with clinically significant disease that were identified, the reduction in over-diagnosis that occurred and the observation that less harm was experienced by those randomised to mpMRI. Our previous PROGENY study also showed that the principle of imaging characterisation followed by diagnostic and research biopsy samples had a high uptake by patients with 80% of men approached agreeing to consent to the study [Linch et al, 2017/18, Annals of Oncology].

# OBJECTIVES

**Cross Sectional Component objective**

To develop a robust baseline risk stratification system for men who are referred with a suspicion of prostate cancer. By risk we mean the probability of harbouring clinically significant prostate cancer.

We aim to explore this risk in relation to characteristics measured in men at baseline. These characteristics will be collected from clinical records, mpMRI images, blood, urine and biopsy tissue samples.

**Longitudinal Component objective**

To create and develop new and improved risk stratifications models for men diagnosed with prostate cancer. By risk we mean the probability of developing cancer, cancer progression, metastasis and death.

We aim to explore this risk in relation to characteristics measured in men at baseline. These characteristics will be collected from clinical records, mpMRI images, blood, urine and biopsy tissue samples.

## Primary Outcome

### **Cross sectional component primary outcome**

Presence of clinically significant prostate cancer confirmed on biopsy, defined as any Gleason pattern 7 or greater.

### Longitudinal Component primary outcome

Time to metastasis and/or prostate cancer related death.

## Secondary Outcomes

- Time to new prostate cancer in men without cancer at baseline
- Time to cancer progression* in men identified with prostate cancer at baseline
- Time to prostate cancer specific death and all-cause death in all men.

** Need for salvage therapy to the prostate following the primary treatment strategy, or, need for systemic therapy for prostate cancer treatment following failure of the primary treatment strategy.*

# STUDY DESIGN

ReIMAGINE Prostate Cancer Risk is a multi-centre, prospective, observational, longitudinal cohort study of men referred to secondary care with a suspicion of prostate cancer or men who are undergoing further tests for prostate cancer staging assessment. Men with a serum PSA level of 20ng/ml or less, whose mpMRI scan has been scored as PIRADS/LIKERT score 3, 4 or 5, and who have been advised and accepted the need for a targeted and systematic prostate biopsy will be invited to enrol.

Men will be recruited across a number of high-volume NHS centres which already have an mpMRI based diagnostic pathway. The study has an initial cross-sectional component where consenting men will be asked to donate blood, urine, imaging files and prostate biopsy tissue for biomarker analysis before or at the time of their standard of care prostate biopsy.

After collection of the cross-sectional biological samples within ReIMAGINE, men will revert to NHS standard of care, attending a routine outpatient appointment for the results of their prostate biopsy outside of the trial setting. Although there are no further planned study visits all men will be consented for healthcare data linkage to measure long-term outcomes including cancer progression, development of metastases and mortality for the longitudinal component of the study.

The ReIMAGINE team aim to recruit 1,000 men with a PIRADs/LIKERT score 3, 4 or 5 who are undergoing an MRI-directed prostate biopsy. Of these 1,000 biopsies, we anticipate that 60% will have any cancer detected on histology (3+3 or greater) giving approximately 400 cases of clinically significant cancer (Gleason 7 or more).

After 12 months of accrual to the study an interim review will be performed by the ReIMAGINE scientific advisory board *(see appendix I for SAB membership)* who will review both accrual rates and cancer incidence with the remit of reviewing and advising on the final recruitment target.

# STUDY SCHEDULE

## Screening and Registration

Potential participants will be identified in a routine secondary care setting of the participating NHS sites by their clinical care team or ReIMAGINE funded staff. Participants may be identified at the point of referral to the hospital, within urology cancer clinics or by other means such as waiting lists or review of case records of the same NHS sites.

First approach of potential participants will predominantly occur via telephone and/or invitation letter conducted or sent by a ReIMAGINE staff member (clinical trials practitioner) following receipt and review of a primary care referral to the recruiting site. As some recruiting sites run a “one-stop” prostate cancer clinic, whereby men are biopsied on the same day as their MRI, the study protocol will include the option to send out a PIS to potential participants and call them to explain the study and discuss any questions they may have, following the clinical team having already explained the clinical standard pathway. As the time window between the MRI (which has to be reported to confirm eligibility) and biopsy (which can only be performed after blood and urine sample collection) is minimal approach by telephone is key to accrual.

If approached via telephone potential participants will be asked for their willingness to receive a patient information sheet (post/email) which will be sent in advance of their clinical appointment. All participants considered for recruitment onto the ReIMAGINE study will be recorded on the study participant screening log. To ensure completeness of data, any patient who receives a patient information sheet for the ReIMAGINE study- either at a face to face consultation, or through the postal service or email- will be included on the study participant screening log.

Consent will also be discussed over the phone and affirmation will be noted both on the eCRF and in medical records (electronic or paper), and on the day that the participant attends clinic for the first visit, wet signature will be obtained, to substantiate affirmation.

Recruiting sites will register fully eligible and consenting patients using a software system (Research Data Collection Service - REDCap) hosted securely by UCL and Phillips (pseudonymised data only). After providing full informed consent, each patient will receive a unique patient ID. An additional separate REDCap database will be hosted securely by University College London (only) to handle identifiable data used for healthcare data linkage purposes.

Following consent, participants will be registered and screened against inclusion/exclusion criteria to confirm eligibility prior to registration (see section 7). Any participant who meets all the inclusion criteria and none of the exclusion criteria will be offered to continue with the study and will be registered. They will attend a baseline visit in order to start collecting baseline measurements.

Eligible men will be invited to consent to the following:

- Agreement to have blood and urine taken for biomarker assessment.
- Agreement for up to 3 additional prostate biopsy cores to be taken at the time of their standard of care diagnostic prostate biopsy.
- Agreement to share standard of care paraffin processed tumour blocks and H&E slides for additional research analysis. These will be returned to NHS sites.
- Donation of anonymised imaging files / data from their baseline MRI scan.
- Agreement to healthcare data linkage through NHS Digital, Public Health England and/or other relevant bodies.

Any patient who provides full informed consent to the study but does not fulfil all other eligibility criteria will be recorded as a screen failure. These screen failure patients will not receive a unique patient ID number.

**Visit 1**

Where participants are found to be eligible the following baseline information and assessments will be recorded prior to registration:

***Demographic and general measurements***

- Medical history
- Demographics
- Family History
- Ethnicity
- Age
- Co-Morbidities
- Medication use

All consented patients who are deemed eligible will have already been advised and/or have a scheduled visit for a prostate biopsy on clinical grounds as standard care. On the day of consent or at the time of the scheduled prostate biopsy participants will have the below interventions:

- Donation of blood will be drawn from men, either via a trained phlebotomist or from the cannula already in situ in preparation for their prostate biopsy.
- Donation of urine. Provided by participant in advance of prostate biopsy.
- Up to three additional biopsy cores will be taken at the time of the standard of care biopsy. Two cores will be taken from the radiologically guided area of suspicion and one control sample from the contralateral non-suspicious area.

**Non-Visit**

After the standard of care prostate biopsy is reported, and in line with participant consent, delegated ReIMAGINE staff members, will request access to the formalin fixed paraffin embedded (FFPE) tissue blocks and H&E (Haematoxylin and Eosin) stained slides from this prostate biopsy.

H & E slides will be anonymised, scanned using high resolution scanners, uploaded to the ReIMAGINE image databank and returned to the NHS pathology department it was requested from.

A tissue technician will acquire slices from the requested FFPE tissue blocks in line with the study SOP and return the block to the hospital pathology department after tissue capture. The acquired slices will be stored in the UCL Cancer Institute laboratory and transported in batches to ReIMAGINE consortium partners.

**Analyses**

Components of blood (white blood cells, plasma, serum) urine and tissue will be subjected to molecular analyses, including genomic, mRNA expression and protein studies by the RE-IMAGINE consortium academic and industry partners. These will include but not be exclusive to next-generation sequencing, whole-genome micro-array expression profiling, immunohistochemistry and immunofluorescence and ELISA tests.

ReIMAGINE Prostate Cancer Risk will be looking to redefine a new clinical metric for significance in relation to prostate cancer (excluding imaging and biomarkers initially).

**Long term healthcare data linkage**

The study will obtain informed consent from participants to collect long term healthcare information from national records, such as the Office for National Statistics, NHS Digital, Public Health England, and/or other applicable NHS information systems, or national databases. No further visits will be required for this activity, but permission will be obtained to receive and hold this information. The research team will collect this healthcare information up to three years after last patient last visit (LPLV) and further funding will be sought to collect this longer term (until death time point should funding be successful).

**Figure 2:** ReIMAGINE Prostate Cancer Risk Trial Flow Diagram

*
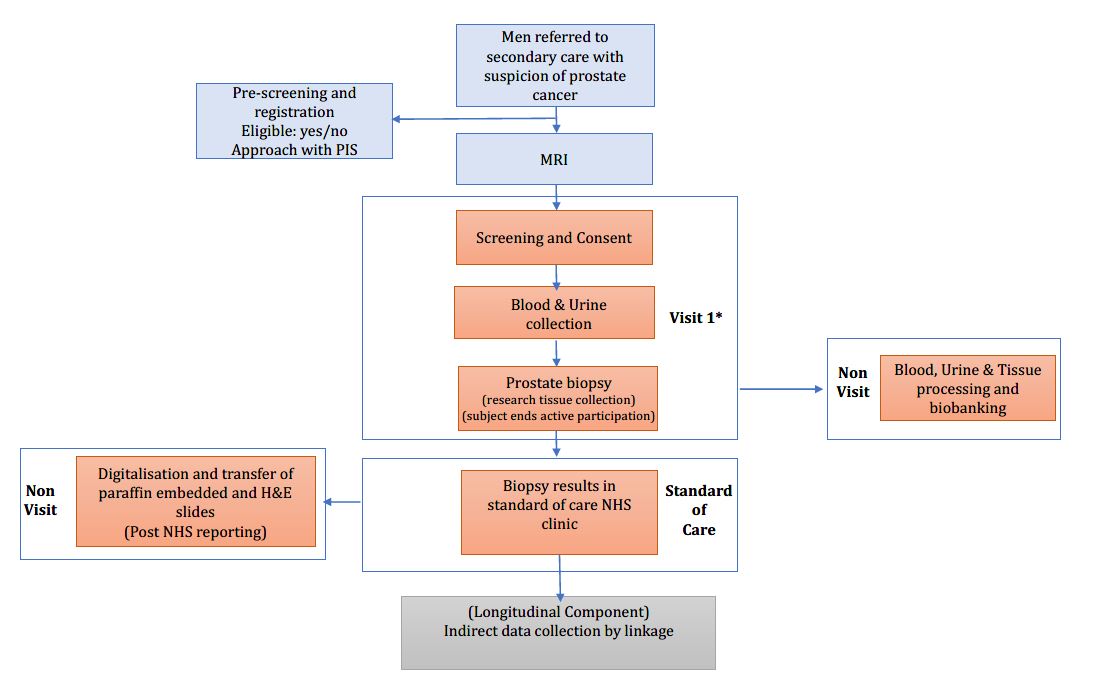
*

** Blood and Urine collection will always occur before the prostate biopsy is performed but may not always be on the same day as the biopsy procedure.*

**Table 1:** Schedule of Assessments

| **Visit** | **Screening & Registration** | **Visit 1*** | **Non-Visit** | **In direct collection** |
| --- | --- | --- | --- | --- |
| Timing of Visit | Day -1 | Day 1 | 3-6 Weeks | n/a |
| Registration | X |  |  |  |
| Medical History | X |  |  |  |
| Eligibility confirmation | X | X |  |  |
| Informed Consent |  | X |  |  |
| PSA | X |  |  |  |
| Research Blood |  | X |  |  |
| Research urine |  | X |  |  |
| Targeted biopsy |  | X |  |  |
| Biopsy results |  |  | X |  |
| FFPE tissue block and H&E slide collection |  |  | X |  |
| Adverse Events review |  | X |  |  |
| Concomitant Medication review (if applicable) | X |  |  |  |
| Healthcare data linkage |  |  |  | X |

** Blood and Urine collection will always occur before the prostate biopsy is performed but may not always be on the same day as the biopsy procedure.*

## Discontinuation/withdrawal of participants

In providing full informed consent to participate in the study, participants are consenting to data collection, screening assessments, sample donation, follow-up and healthcare data linkage.

Sites will be required to complete a protocol deviation form if participants are unable to provide:

- < 10ml of blood. (1x 10ml EDTA tube)
- < 10ml urine
- < 1 prostate tissue core

A participant may be withdrawn from the study whenever continued participation is no longer in the participant’s best interests, but the reasons for doing so must be recorded. Reasons for discontinuing the study may include:

- - intercurrent illness
  - patients withdrawing consent
  - Persistent non-compliance to protocol requirements.

The decision to withdraw a participant from the study will be recorded in the eCRF and medical notes. If a participant explicitly states that they do not wish to contribute further data to the study their decision must be respected and recorded in the eCRF and medical notes. Should any participant withdraw from the study, any information that has already been obtained will be kept, in line with the Data Protection Act (2018) and General Data Protection Regulations (GDPR).

## Definition of End of Study

**For each man**

Following consent and sample collection (blood, urine, prostate tissue), men will revert back to the NHS standard of care pathway. Men will be booked into a routine NHS clinical appointment to receive the result of their standard of care prostate biopsy. Men will undergo follow-up for 4-8 weeks to gather information from their medical records (i.e. the result of the biopsy) at which time active data collection for the cross-sectional component will end. Men will then transition to the longitudinal component of the study and will be followed up via data linkage (initial 3-year period).

For the cross-sectional component, end of study is defined as database lock.

For the longitudinal component end of study is defined as database lock.

# CONSENT

Before any study-related procedures are performed (blood, urine or tissue capture), the Principal Investigator or his/her delegate will obtain full written informed consent from a participant. Participants can withdraw consent and decide not to remain in the study at any time without their care being affected.

Participants considered potentially eligible will be approached by a member of the ReIMAGINE team (clinical trials practitioner) or routine clinical team. There are several approach points when this may occur, all of which must be before the prostate biopsy is performed:

- Via telephone upon receipt of a primary care referral to the recruiting centre.
- In clinic before the MRI has been performed and/or reported.
- In clinic after the MRI has been reported and the potential participant has been clinically advised to have a prostate biopsy (which may occur later that day).
- Via telephone after the MRI has been reported and the potential participant has been clinically advised to have a prostate biopsy (which has been or is in the process of being scheduled).

Potential participants will be provided with verbal and written details about the study (ReIMAGINE detailed Patient Information Sheet/Informed Consent Form [PIS/ICF]) or sent the relevant material in the post. This will include detailed information about the rationale, design and activities of the study. Participants will also be provided with a contact point (ReIMAGINE dedicated CTP) where he may obtain further information about the study.


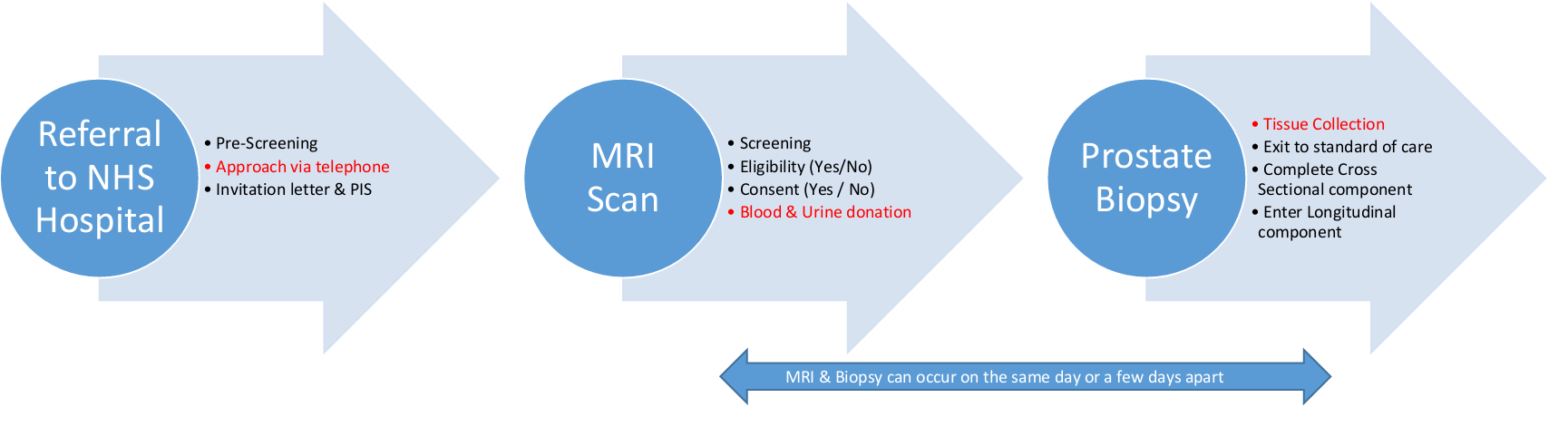


It is the responsibility of the Principal Investigator (PI), or a person delegated by the Investigator to obtain written informed consent from each participant prior to any study specific procedures, following adequate explanation of the aims, methods, anticipated benefits and potential hazards of the study.

The person taking consent will be suitably trained, qualified, experienced and competent to participate according to the ethically approved protocol, principles of Good Clinical Practice (GCP) and Declaration of Helsinki 1996.

Anyone other than the PI who is taking consent will have been delegated this duty by the PI on the ReIMAGINE study delegation log. Where this task has been delegated to another member of the team, the PI retains overall responsibility for the informed consent of participants.

It must be recorded in the medical notes when the patient information sheet (PIS) has been given to a participant.

Following the provision of study information, participants will have as long as they need to consider participation (within the limitations of study duration) and will be given the opportunity to discuss the study with their family and other healthcare professionals before they are asked whether they would be willing to take part. Where participants wish, and in cases of participants having to travel a long distance to access a study site, they will be able to provide consent during the same visit that the Patient Information Sheet was provided or be given the option to provide telephone consent. Alternatively, they may return for a separate visit to provide consent, if this is suitable for the participant.

The Investigator or designee must explain that participants are under no obligation to enter the study and they are not required to provide a reason. It must be explained that they can withdraw at any time during the study, without having to give a reason and without prejudicing his further treatment.

No study-specific procedures will be conducted prior to the participant providing full informed consent.

A copy of the signed informed consent form will be given to the participant. The original signed form will be retained in the study file at site and a copy placed in the medical notes. The PIS and consent form will be reviewed and updated where necessary throughout the study (e.g. where new safety information becomes available) and participants will be re-consented as appropriate. It is the responsibility of the PI to ensure this is done in a timely manner.

**Loss of capacity following informed consent**

Loss of mental capacity of a participant after giving informed consent for the study is expected to be a rare occurrence. In this case no further study procedures or data collection will occur. Any data collected up to the point of withdrawal will be kept on record and used in the study analysis.

# ELIGIBILITY CRITERIA

## Inclusion Criteria

- Any man with PSA 20 or less *(value recorded <12 months before study entry)*
- Men who have undergone a prostate MRI as a standard NHS diagnostic work-up.
- MRI lesion conforming to Likert/PIRADS 3, 4 or 5
- Radiological stage T3b or less
- Clinical or radiological stage N0 and M0
- No anti-androgen exposure in the preceding 6 months (5-alpha reductase inhibitors permitted)
- No prior treatment for prostate cancer (chemical, biological, ablative, surgical, radiotherapy)
- Previous TURP is permitted
- Willing and able to provide written informed consent.

## Exclusion Criteria

- Men unable to donate tissue, blood or urine.
- Previous prostate cancer treatment
- Previous prostate biopsy <12 months from date of the mpMRI scan used to assess study eligibility (scoring PIRADs/LIKER 3, 4 or 5).

# RECRUITMENT

All eligible participants referred to secondary care with a suspicion of prostate cancer will be considered for screening. Potential participants will be primarily identified at the point of referral or during routine secondary care urology cancer clinics of the participating NHS sites by their clinical care team or REIMAGINE funded staff. Alternatively, participants may be identified by other means such as waiting lists or review of case records).

All screening and formal eligibility assessments will subsequently take place in the same NHS clinics, dedicated research clinics or on the surgery ward (in preparation for their biopsy) of the study sites.

Research sites will be required to have obtained local, ethical and management approvals and undertake a site initiation meeting prior to the start of recruitment into the study.

# STATISTICAL METHODS

**Sample Size Calculation**

Men suspected of having prostate cancer will be offered an MRI as part of usual care. Our aim is to recruit men with an MRI lesion scoring 3, 4 or 5 who undergo a biopsy.

The ReIMAGINE team will approach approximately 1,777 men with the study PIS.

Of the 1,777 men approached, 1,333 (75%) based on current literature will be expected to be recorded with PIRADs/LIKERT 3, 4 or 5 and advised to undergo a biopsy. The 444 men with PIRADS/LIKERT 1 or 2 will be excluded from taking part in ReIMAGINE Prostate Cancer Risk.

1,333 men will be offered an MRI-directed biopsy with some additional systematic tissue sampling from the non-MRI-directed areas. We expect 25% will reject biopsy or entry to the study which means 1,000 will have the biopsy procedure after consenting to ReIMAGINE Prostate Cancer Risk.

Of these 1,000 biopsies, we anticipate that 60% will have any cancer detected on histology (Gleason 3+3 or greater) yielding 600 cases of cancer and of these we expect about 396 (66%) will have clinically significant cancer i.e. any Gleason pattern 7 or greater.

Therefore, for the cross-sectional component of the analysis we anticipate 600 cases of cancer and 400 cases of no cancer. We will have 396 cases of clinically significant cancer and 604 cases of no cancer or insignificant cancer.

**Statistical Analysis**

All statistical analyses will be conducted according to pre-specified statistical analysis plans that will be agreed prior to the inspection of any outcome data. Logistic regression modelling will be used to analyse the factors associated with presence of clinically significant cancer. Cox regression modelling (or other appropriate survival methods if there is evidence of non-proportional hazards) will be used to analyse the factors associated with time to new cancers, progressions, metastases and deaths. Initially a list of pre-specified factors will be agreed and these will be analysed in the models using a stepwise approach to identify those that are independently associated with the outcome.

In addition to the simple frequentist analysis approach, a more sophisticated analysis will be conducted. We will first apply the SaddlePoint Signature multivariate Bayesian regression pipeline, which was designed specifically for regression with high-dimensional covariates, in order to generate (i) optimal predictive covariate selections (suppressing false positive associations), (ii) quantitative characteristics of true associations (including covariate interactions, if relevant), (iii) personalised multivariate risk signatures, and (iv) quantifiers of the degree of outcome predictability. This will be done both for the full covariate set and for the individual covariate streams (e.g. imaging, genomic, phenotypic), allowing us to compare the outcome prediction power of different information sources, and their potential synergy. The most recent version of the pipeline (version 2.8.6), uses advanced overfitting correction protocols (based on the replica method) and robust internal validation processes. The risk scores for the separate streams will provide intuitive visualisations of the heterogeneity of the patient cohort. In a second stage we will apply Bayesian multivariate latent class analysis, using the source-specific risk signatures computed in the first stage as meta-covariates, and assess whether and how the cohort should be further stratified into subgroups with distinct associations and/or base hazard rates.

# PATIENT AND PUBLIC INVOLVEMENT (PPI)

The ReIMAGINE project includes a work strand which focusses solely on PPI engagement. This work strand is led by Mr Steve Tuck a prostate cancer survivor, and the ReIMAGINE PPI representative and Dr Mieke Van Hemelrijck.

The clinical investigators have discussed the design of the study including number of visits and outcome measures, with Mr Steve Tuck. There will be PPI representation on the TSC. All patient facing documents will be reviewed by the PPI representative and he will be called upon to contribute to the reporting of the study report and dissemination of research findings.

# FUNDING AND SUPPLY OF EQUIPMENT

The study is jointly funded by the Medical Research Council (£5.18M GBP) and Cancer Research UK (£1M GBP) on grant award MR/R014043/1, funding letter dated 19 February 2018. Grant funded clinical trial practitioners and tissue technicians will undertake the majority of the research activity resulting in minimal impact to NHS resources.

The study funding has been reviewed by the UCL/UCLH Joint Research Office, and deemed sufficient to cover the requirements of the study. NHS treatment costs will be supported via standard NHS commissioning routes and service support activity via Local Clinical Research Networks.

The management of the research will be covered by UCL insurance for negligent harm.

UCL insurance provides cover for negligent harm arising from the design of the research.

The NHS indemnity scheme will apply for negligent harm arising from the conduct of the research.

The study is supported by multiple external partners by way of in-kind contributions to biomarker analysis, imaging analysis and database hosting. The ReIMAGINE consortium will share anonymised MRI images and biological samples with multiple external partners, who will analyse these samples against their biomarker(s) or imaging software. Each external partner will sign the consortium agreement which details the in-kind contributions they will make to the consortium.

# DATA HANDLING AND MANAGEMENT

ReIMAGINE Prostate Cancer Risk will have two separately managed and hosted databases to handle identifiable and pseudonymised data.

**Pseudonymised data**

Data collected in the study will include baseline clinical and demographic data, detailed histology and imaging. Such patient data shall be accessed, with full informed consent, using hospital-specific software systems and paper medical notes.

Source data will be entered onto the study database from each recruiting centre using an electronic case report form (eCRF). The Chief Investigator (CI) is responsible for reviewing all information collected on patients enrolled in the study for completeness and accuracy. The delegation log will identify and name all those personnel with responsibility for both data collection and handling and will include further information on the access rights of each delegate to the data warehouse.

The data warehouses, which are hosted and managed securely by UCL and Philips in the EU, will be safeguarded against unauthorised access with established security procedures. Additional maintenance will include regular back-ups and upkeep of related software files. Patients will not be identifiable in the study database but will be identified by unique patient ID numbers assigned at the point of recruitment into the study.

The identification, screening and enrolment logs which will link participant identifiable data to the pseudonymised patient ID number will also be held locally by each study site. Physical copies of these logs will be stored in locked filing cabinets and electronic versions held on password protected NHS Trust computers.

It is the responsibility of each study site to maintain a file of essential study documentation (Investigator Site File), and to maintain their file of essential study documentation on site during the study and thereafter at the designated archive facility.

The Chief Investigator, Philips and delegates are responsible for the daily management of the data warehouse. The following guidelines will be strictly adhered to:

Any patient data stored on the software system hosted by Philips will be pseudonymised.

All anonymised data will be password-protected, with differing levels of access agreed for each delegate prior to study initiation.

Database management methods will be documented according to scientific method standards which will contain enough detail to theoretically allow independent researchers to repeat the experiment. The data warehouse software’s provided by UCL and Philips include various features to ensure data quality including maintenance of audit trail, allowance of custom validation on all data and the ability for users to raise data query requests and search facilities to identify missing data or validation failure.

Copies of the final locked database with documentation of variables, coding and data cleaning before statistical analysis will be stored by the database manager. The database manager will run queries to extract the data. The data will be extracted in tables and provided to the researchers in excel and CVS format. Data documentation will be supported by the Statistical Master File which will include: details of statistician consistency checks; documentation of import of statistical data; statistical package; location of statistical program files. All statistical analysis programs will be fully documented.

The ReIMAGINE consortium includes data sharing agreements with a number of academic and commercial partners. All data released from the consortium will appear in an anonymised format. Access to the shared data repository will be restricted and controlled by the database team based at University College London in partnership with King’s College London. The shared data repository infrastructure will be provided by UCL and Philips. The Philips team will have limited access to data as agreed in the data sharing contract signed by the ReIMAGINE lead institution. All commercial and academic partners involved in the ReIMAGINE consortium have signed and agreed specific data sharing contracts – all based on pseudonymised data.

After completion of the ReIMAGINE study, the data warehouse (hosted by Philips) will be transferred securely from Philips to University College London for on-going analysis of secondary outcomes. Data transfer will occur using end to end encryption.

All data will be handled in accordance with the Data Protection Act 2018 and General Data Protection Guidance (2018).

**Identifiable Data**

Patients will be asked to give their consent to provide identifiable information such as their name, address, date of birth and NHS number at point of entry to the study. This information will be stored securely by University College London in the United Kingdom. This identifiable data will be used to collect healthcare information on men from national records, such as the Office for National Statistics, NHS Digital, Public Health England, and other applicable NHS information system, or national databases. This process will be clearly explained to patients in the patient information sheet provided prior to consent. The Chief Investigator agrees to hold such identifiable information, alongside the assigned unique patient ID number, securely on servers hosted and maintained by UCL. This server is fire-wall protected and provides auto failover redundancy in the event of hardware failure. The data backup schedule follows standard full backups. The physical location of this server is protected by CCTV and security door access. All records will be retained by the CI according to the International Conference on Harmonisation (ICH) or local regulations; all study documentation will be retained for 20 years after the study end date unless advised otherwise by the sponsor.

Access to the identifiable patient data held securely at University College London will be restricted to named personnel only. Only the data controller and named members of the data management team will be granted access to the identifiable patient data held securely at University College London.

# MATERIAL/SAMPLE STORAGE

In the study blood, urine and prostate tissue cores will be collected from patients in accordance with the patient consent form and patient information sheet and shall include all tissue samples or other biological materials and any derivatives, portions, progeny or improvements as well as all patient information and documentation supplied in relation to them. Samples will be processed, stored and disposed in accordance with all applicable legal and regulatory requirements, including the Human Tissue Act 2004 and any amendments thereafter.

Biological samples will be stored under HTA licence 12055 and shipped periodically to academic and commercial partners in the EU, North America and Australia for analyses. These partners will be part of the ReIMAGINE consortium and have appropriate material transfer agreements (MTA’s) in place before shipment of any samples from the central laboratory at UCL. A list of partners is detailed in appendix III.

Access to any human samples requires a request and approval by the biological research group that will maintain scientific rigour and prioritisation of material. Samples are not to be processed and/or transferred other than in accordance with the patients’ consent.  After ethics approval for the study has expired, the blood, urine and tissue samples will be disposed of in accordance with the Human Tissue Act 2004 and any amendments thereto or transferred to a licensed tissue bank.

## Blood Sample Collection

- Blood samples will be collected by trial employed clinical trial practitioner in advance or on the day a MRI guided biopsy is taken. This could be in a dedicated clinic, on the ward or intraoperatively during the biopsy procedure (when a cannula is already in situ). The patients will receive a copy of their signed consent form and the Patient Information Sheet.
- A minimum volume of 50ml will be requested, with a maximum volume of 100ml in men who are able and willing to provide this.
- The blood samples will be processed by a CTP or a Technician using the hospital’s laboratory facilities. For detailed information on preparation, storage and shipment of blood samples please refer to the ReIMAGINE Prostate Cancer Risk Laboratory Manual.
- Once the whole blood has been processed, it will be logged into FreezerPro database. No patient identifiable information will be entered onto the database and all patient information will be pseudonymised. All samples will be stored temporarily onsite at -80C. At regular intervals the samples will be sent using a courier to:

**Shipping Address:**

LAB 205, UCL Cancer Institute

Paul O’Gorman Building

72 Huntley Street

London WC1E 6DD

- Once received at the laboratory, the samples will be stored in a secure location in -80 freezers. The temperature will be monitored using T-scan software and the freezers will have a CO2 back up system attached.
- When required, the aliquots will be sent to consortium partners for analyses in line with study SOP’s and MTA’s.

## Urine sample collection

- Urine samples will be collected by trial employed clinical trial practitioner in advance or on the day a MRI guided biopsy is taken. This could be in a dedicated clinic or on the ward but will always occur in advance of the biopsy.
- Urine samples will be provided by participants into urine collection bottles and processed according to ReIMAGINE Prostate Cancer Risk Lab manual.
- The samples will be logged into the FreezerPro database and stored temporarily at the collection sites.
- At the regular intervals the samples will be shipped using a courier to:

**Shipping Address:**

LAB 205, UCL Cancer Institute

Paul O’Gorman Building

72 Huntley Street

London WC1E 6DD

- Once received at the laboratory, the samples will be stored in a secure location in -80 freezers. The temperature will be monitored using T-scan software and the freezers will have CO2 back up system attached.
- When required the aliquots will be sent to consortium partners for analyses in line with study SOP’s and MTA’s.

## Prostate tissue Collection

- In addition to diagnostic tissue collected for FFPE processing, up to 3 cores of fresh tissue (standard prostate biopsy cores) will be collected and frozen intraoperatively. For detailed information on preparation, storage and shipment of tissue samples please refer to the ReIMAGINE Prostate Cancer Risk Laboratory Manual.
- Once the samples are collected, they will be logged into FreezerPro and temporarily stored at the hospital sites at -80.

The samples will be shipped a regular intervals using courier to:

**Shipping Address**

LAB 205, UCL Cancer Institute

Paul O’Gorman Building

72 Huntley Street

London WC1E 6DD

- Once received at the laboratory, the samples will be stored in a secure location in -80 freezers. The temperature will be monitored using T-scan software and the freezers will have CO2 back up system attached.
- When required tissue samples will be sent to consortium partners for analyses in line with study SOP’s and MTA’s.

## Paraffin and H&E slide collection

In addition to the samples collected at visit 1, all sites will send H&E slides and FFPE tissue blocks to Professor G Attard’s laboratory (facilitated by the ReIMAGINE clinical trial practitioners).

H & E slides will be anonymised, scanned using high resolution scanners and uploaded to the ReIMAGINE image warehouse. Scanned H&Es could be subjected to additional central pathology assessment and morphological features captured using computer-based algorithm to identify H&E based biomarkers for improved patient risk stratification. Scanned H&Es could also be shared with partners to integrate with analyses of scans and molecular biomarkers.

A tissue technician will also acquire slices from the requested FFPE tissue blocks in line with the study SOP. The acquired slices will be stored in the Attard laboratory and transported in batches to consortium partners for analyses in line with study SOP’s and MTA’s. Additional sections will be subjected to molecular analyses, including genomic, mRNA expression and protein studies by the RE-IMAGINE consortium academic and industry partners.

Both H&E slides and FFPE tissue blocks will be returned to the relevant NHS pathology department.

Instructions on postage and packing will be shared with sites at SIV.

## Imaging databank

Clinical trial practitioners will request pseudonymised DICOM (Digital Imaging and Communications in Medicine) images from the standard of care prostate MRI each recruited participant had before entry to the study. These anonymised images will be uploaded to the CRUK funded National Cancer Imaging Translational Accelerator (NCITA) image repository, hosted by UCL.

A copy of these pseudonymised images will be transferred via the XNAT platform to the ReIMAGINE data warehouse hosted by Philips in the EU. Both platforms have strict safeguards against unauthorised access with established security procedures.

# PEER AND REGULATORY REVIEW

The study has been peer reviewed in accordance with the requirements outlined by UCL/UCLH.

The Sponsor considers the procedure for obtaining funding from the Medical Research Council to be of sufficient rigour and independence to be considered an adequate peer review.

The study was deemed to require regulatory approval from the Regional Ethics Committee and Health Research Authority. Each approval will be obtained before the study commences.

# ASSESMENT AND MANAGEMENT OF RISK

The table below summarise the risks and mitigations of all test above standard of care that are being performed:

| **Intervention** | **Potential risk** | **Risk Management** |
| --- | --- | --- |
| Tissue donation | Biopsies will be performed as part of standard of care, additional cores will be performed for research. A biopsy may result in bleeding, bruising and infection. | Performed by trained urologists, following trust standard operational procedures.  Maximum of 3 cores taken which should infer minimal burden. |
| Blood donation | Bruising, Pain, Bleeding and Infection | Performed by a trained phlebotomist, following trust standard operating procedures.  Or can be taken when cannula already in situ in preparation for prostate biopsy to avoid necessity for additional skin puncture. |
| Urine donation | None | Will be performed by the participant. |

# RECORDING AND REPORTING OF EVENTS AND INCIDENTS

This study is not expected to have any unexpected adverse events as the procedures that patients will receive form part of standard of care. Cross-sectional blood, urine and tissue samples will be collected at which point active follow-up to this component will end. Expected adverse events that occur thereafter may be due to the standard of care biopsy that is performed at the point the cross-sectional component ends.

Thus, this study will not document adverse events in particular however; any events that do occur should be recorded in the patient’s medical notes and will be reviewed during monitoring visits.

## Recording adverse events

All adverse events will be recorded in the medical records in the first instance.

As an observational trial this study involves no specific interventions and all participants will undergo standard of care investigations, although additional biopsies and a blood draw is required. No risk is expected from giving a urine sample.

A list of expected AEs which do not need reporting except for in the medical notes include the following. Severity can escalate to serious:

- Pain
- Blood in the urine
- Blood in the semen
- Blood in the stool or back passage
- Erectile dysfunction
- Urinary incontinence
- Urinary tract infection
- Fevers

## Procedures for recording and reporting Serious Adverse Events

As ReIMAGINE Prostate Cancer Risk is not an interventional study, no SAEs related to the treatment of prostate cancer or any other treatment related SAEs will be collected. These events must continue to be recorded in the participant’s medical records.

Death is a study primary end point, therefore deaths must be reported on a death form, and not as an SAE.

# PROTOCOL DEVIATIONS AND NOTIFICATION OF PROTOCOL VIOLATIONS

A deviation is usually an unintended departure from the expected conduct of the study protocol/SOPs, which does not need to be reported to the sponsor. The CI will monitor protocol deviations.

A protocol violation is a breach which is likely to effect to a significant degree –

(a) the safety or physical or mental integrity of the participants of the study; or

(b) the scientific value of the study.

The CI and sponsor will be notified immediately of any case where the above definition applies during the study conduct phase, by completion of a PD/PV eCRF.

# TRUST INCIDENTS AND NEAR MISSES

An incident or near miss is any unintended or unexpected event that could have or did lead to harm, loss or damage that contains one or more of the following components:

a. It is an accident or other incident which results in injury or ill health.

b. It is contrary to specified or expected standard of patient care or service.

c. It places patients, staff members, visitors, contractors or members of the public at unnecessary risk.

d. It puts the Trust in an adverse position with potential loss of reputation.

e. It puts Trust property or assets in an adverse position or at risk.

Incidents and near misses must be reported to the Trust through DATIX as soon as the individual becomes aware of them.

A reportable incident is any unintended or unexpected event that could have or did lead to harm, loss or damage that contains one or more of the following components:

1. It is an accident or other incident which results in injury or ill health.
2. It is contrary to specified or expected standard of patient care or service.
3. It places patients, staff members, visitors, contractors or members of the public at unnecessary risk.
4. It puts the Trust in an adverse position with potential loss of reputation.
5. It puts Trust property or assets in an adverse position or at risk of loss or damage.

# MONITORING AND AUDITING

The Chief Investigator will ensure that there are an adequate number of monitoring activities of high quality, conducted by the study team. Such monitoring activities will include adherence to the protocol, procedures for consenting and ensuring adequate data quality.

The Chief Investigator will inform the sponsor should he/she have concerns which have arisen from monitoring activities, and/or if there are problems with oversight/monitoring procedures.

Risk will be assessed on an ongoing basis and adjustments made accordingly.

The degree of monitoring will be proportionate to the risks associated with the study.

A study specific oversight and monitoring plan will be established for ReIMAGINE Prostate Cancer Risk. The study will be monitored in accordance with the agreed plan.

# TRAINING

Grant funded staff will be trained to comply with standard operating procedures for sample collection, processing and storage.

No other additional specific training is required for the ReIMAGINE WS 1 study.

All sites will attend an SIV prior to patients being consented, and GCP certificates will be kept up to date (expire after 2 years). An attendance log of all staff present at SIV will be held on file and training would be cascaded to new staff, by individuals with training responsibly delegated to them by the site PI, this will be noted on each site’s delegation log. All new members of staff training to support ReIMAGINE Prostate Cancer Risk will be noted on sites training logs. Appropriate training records will be maintained in the investigator study files.

# INDEMNITY ARRANGEMENTS

University College London holds insurance against claims from participants for harm caused by their participation in this clinical study. Participants may be able to claim compensation if they can prove that UCL has been negligent. However, if this clinical study is being carried out in a hospital, the hospital continues to have a duty of care to the participant of the clinical study. University College London does not accept liability for any breach in the hospital’s duty of care, or any negligence on the part of hospital employees. This applies whether the hospital is an NHS Trust or otherwise.

# STUDY GOVERNANCE

Research sites will liaise with the ReIMAGINE Trial Management Group (TMG) for advice and support on study set up and operation, and submission of study data. In turn, the ReIMAGINE Team (SITU) will be responsible for data chasing.


## Responsibilities

The CI is responsible for the design, management and reporting of the study.

The Surgical & interventional Trials Unit will have responsibility for overall conduct of the study in accordance with the NHS Research Governance Framework and sponsors and SITU SOPs.

The responsibility for ensuring clinical management of the participants is conducted in accordance with the trial protocol and ultimately remains with the PI at each site.

## Operational Structure

Chief Investigator (CI): as defined by the NHS Research Governance Framework, is responsible for the design, conduct, coordination and management of the study.

Trial sponsor – University College London (UCL). The sponsor is responsible for ensuring before a study begins that arrangements are in place for the research team to access resources and support to deliver the research as proposed and allocate responsibilities for the management, monitoring and reporting of the research. The Sponsor also has to be satisfied there is agreement on appropriate arrangements to record, report and review significant developments as the research proceeds, and approve any modifications to the design.

## Oversight/ Study Monitoring Groups

**The Executive Board (EB)** will have overall responsibility for overseeing implementation of the project and for overseeing reporting to the funder as required by the Award Terms and Conditions. The EB is the senior decision-making body of the consortium in respect of the project. The EB will meet at least twice a year as a group. Please see appendices for membership.

**The Steering Committee (SC)** will have responsibility for monitoring and overseeing overall progress of the Project and for providing informed guidance in respect of the same to the EB. The SC will offer an international stakeholder perspective and a representative of the SC will meet annually with the Executive Board. They will receive six-monthly status reports. The SC will meet annually. Initial membership details of the SAB is listed in the appendices.

**The Scientific Advisory Board (SAB)** will provide independent advice to the EB & SC. Its members will be external to the project team. They will receive six-monthly status reports. The SAB will meet annually and a representative will be invited to the EB meetings at least once per year. Initial membership details of the SAB is listed in the appendix.

**Biological Research Committee (BRC)** will have responsibility for developing and refining rules for access to collaboration materials, clinical samples generated/collected under the Project and/or data, including rules for access by external applicants, ensuring such rules comply with the Funding Conditions plus all relevant Funder guidelines. The BRC will initially assess the addition of other organisations that may wish to join the Consortium after the Effective Date and make recommendations based on their findings to the EB. The BRC will meet at least once per year as a group and a representative will meet annually with the EB. The BRC will be accountable to the EB. Initial membership details of the BRC are listed in the appendix.

**Trial Management Group (TMG)** The TMG is responsible for the day-to-day running of the trial and can make decisions appropriate to this role. The TMG will meet monthly face to face or via teleconference and create a direct link with staff working at the recruiting NHS sites.

# ARCHIVING

UCL and each participating site recognise that there is an obligation to archive study-related documents at the end of the study (20 years). The Chief Investigator confirms that he will archive the study master file at University College London for the period stipulated in the protocol and in line with all relevant legal and statutory requirements. The Principal Investigator at each participating site agrees to archive his/her respective site’s study documents for 20 years and in line with all relevant legal and statutory requirements.

# PUBLICATION AND DISSEMINATION POLICY

The success of the study depends upon the collaboration of all parties. For this reason, credit for the main results will be given to all those who have collaborated in the study, through authorship and contributions. Uniform requirements for authorship for manuscripts submitted to medical journals will guide authorship allocations. These state that authorship credit should be based only on substantial contribution to:

- Conception and design, or acquisition of data, or analysis and interpretation of data,
- drafting the article or revising it critically for important intellectual content,
- Final approval of the version to be published and,
- That all these conditions must be met ([www.icmje.org](http://www.icmje.org)).
- A publication policy will be written and agreed by the Steering Committee. Presentations and publications arising directly from the pre-planned analyses will be the responsibility of the Steering Committee.
- Other members of the medical and scientific community will be encouraged to submit requests for new analysis of the data set. However, the raw data will remain in the custodianship of the Trials Operations Office, apart from the transfer of anonymised data to the Study Statistician for pre-determined analyses.
- The patient information leaflet sign-posts all participants to study website at, <https://www.reimagine-pca.org> and the SITU website at, [www.ucl.ac.uk/surgery/research/surgical-interventional-trials-unit-situ](http://www.ucl.ac.uk/surgery/research/surgical-interventional-trials-unit-situ) where all publications will be made publically available.

# REFERENCES

Ahmed HU, El-Shater Bosaily A, Brown LC, Gabe R, Kaplan R, Parmar MK, Collaco-Moraes Y, Ward K, Hindley RG, Freeman A, Kirkham AP, Oldroyd R, Parker C, Emberton M; PROMIS study group. [Diagnostic accuracy of multi-parametric MRI and TRUS biopsy in prostate cancer (PROMIS): a paired validating confirmatory study.](https://www.ncbi.nlm.nih.gov/pubmed/28110982)

Lancet. 2017 Feb 25;389(10071):815-822. doi: 10.1016/S0140-6736(16)32401-1. Epub 2017 Jan 20.

PMID:28110982

Baco E, Ukimura O, Rud E, Vlatkovic L, Svindland A, Aron M, Palmer S, Matsugasumi T, Marien A, Bernhard JC, Rewcastle JC, Eggesbø HB, Gill IS.

Magnetic resonance imaging-transectal ultrasound image-fusion biopsies accurately characterize the index tumor: correlation with step-sectioned radical prostatectomy specimens in 135 patients.

Eur Urol. 2015 Apr;67(4):787-94. doi: 10.1016/j.eururo.2014.08.077. Epub 2014 Sep 17. PubMed PMID: 25240973.

Bill-Axelson A, Holmberg L, Garmo H, Taari K, Busch C, Nordling S, Häggman M, Andersson SO, Andrén O, Steineck G, Adami HO, Johansson JE. [Radical Prostatectomy or Watchful Waiting in Prostate Cancer - 29-Year Follow-up.](https://www.ncbi.nlm.nih.gov/pubmed/30575473) N Engl J Med. 2018 Dec 13;379(24):2319-2329. doi: 10.1056/NEJMoa1807801. PMID:30575473

Fraser M, Sabelnykova VY, Yamaguchi TN, et al. Genomic hallmarks of localized, nonindolent prostate cancer. Nature. 2017 Jan 19;541(7637):359-364. doi: 10.1038/nature20788.

Epub 2017 Jan 9. PMID:28068672

Grönberg H, Adolfsson J, Aly M, et al. Prostate cancer screening in men aged 50-69 years (STHLM3):

a prospective population-based diagnostic study. Lancet Oncol. 2015 Dec;16(16):1667-76. doi:

10.1016/S1470-2045(15)00361-7. Epub 2015 Nov 10. PMID:26563502

Mariam Jamal-Hanjani, Gareth A. Wilson, Nicholas McGranahan, Nicolai J. Birkbak, Thomas B.K. Watkins, Selvaraju Veeriah, Seema Shafi, Diana H. Johnson, Richard Mitter, Rachel Rosenthal, Max Salm, Stuart Horswell, M.Math., et al., for the TRACERx Consortium
Tracking the Evolution of Non–Small-Cell Lung Cancer. N Engl J Med June;2017; 376:2109-2121 DOI: 10.1056/NEJMoa1616288. PMID: 28445112

Kasivisvanathan V, Rannikko AS, Borghi M, Panebianco V, Mynderse LA, Vaarala MH, Briganti A, Budäus L, Hellawell G, Hindley RG, Roobol MJ, Eggener S, Ghei M, Villers A, Bladou F, Villeirs GM, Virdi J, Boxler S, Robert G, Singh PB, Venderink W, Hadaschik BA, Ruffion A, Hu JC, Margolis D, Crouzet S, Klotz L, Taneja SS, Pinto P, Gill I, Allen C, Giganti F, Freeman A, Morris S, Punwani S, Williams NR, Brew-Graves C, Deeks J, Takwoingi Y, Emberton M, Moore CM; PRECISION Study Group Collaborators. [MRI-Targeted or Standard Biopsy for Prostate-Cancer Diagnosis.](https://www.ncbi.nlm.nih.gov/pubmed/29552975) N Engl J Med. 2018 May 10;378(19):1767-1777. doi: 10.1056/NEJMoa1801993. Epub 2018 Mar 18.

PMID:29552975

# APPENDIX I: Oversight/Study monitoring Group Membership

**Executive Committee Membership**

PI (C) and WS Leads

Patient rep

Statistician

Project Manager

Senior Trial Coordinator

Representative of each Commercial Party

**Scientific Advisory Board Membership**

Four internationally recognised experts in fields relevant to The Project, who are able to provide independent and informed guidance to the Executive Board.

Caroline Dive

Eytan Domany

Malcolm Mason (C)

Anwar Padhani

**Steering Committee Membership**

Eric Aboagye

Richard Kaplan (c)

Chris Parker

Peter Parker

**Biological Research Committee Membership**

Gert Attard (C)

Charlotte Bevan

Paul Boutros

Andrew Feber

Hayley Whitaker

**Trial management Group Membership**

Hashim Ahmed (C)

Mark Emberton

Louise Brown

Mieke Van Hemelrijck

Gerhardt Attard

Aida Santaolalla

Chris Brew-Graves

Neil McCartan


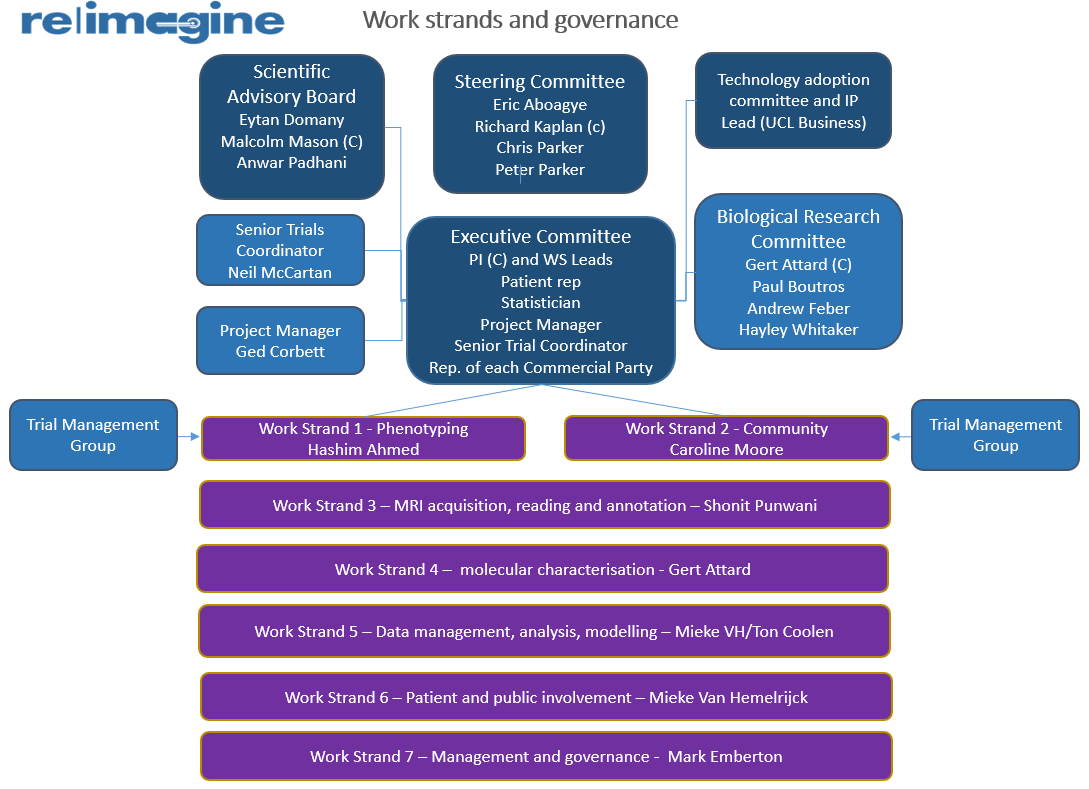


# APPENDIX II: Schedule of assessments

| **Visit** | **Screening & Registration** | **Visit 1*** | **Non Visit** | **In direct collection** |
| --- | --- | --- | --- | --- |
| Timing of Visit | Day -1 | Day 1 | 3-6 Weeks | n/a |
| Registration | X |  |  |  |
| Medical History | X |  |  |  |
| Eligibility confirmation | X | X |  |  |
| Informed Consent |  | X |  |  |
| PSA | X |  |  |  |
| Research Blood |  | X |  |  |
| Research urine |  | X |  |  |
| Targeted biopsy |  | X |  |  |
| Biopsy results |  |  | X |  |
| FFPE tissue block and H&E slide collection |  |  | X |  |
| Adverse Events review |  | X |  |  |
| Concomitant Medication review (if applicable) | X |  |  |  |
| Healthcare data linkage |  |  |  | X |

# APPENDIX III: ReIMAGINE Consortium partners

The fifteen parties collaborating in the ReIMAGINE Consortium (at Project Start).

(1) University College London

(2) King’s College London

(3) Imperial College London

(4) Affidea BV

(5) Chronix Biomedical Inc.

(6) GenomeDx Biosciences Inc.

(7) Image Analysis Limited

(8) Maxwell

(9) MDNA Life Sciences

(10) MDx Health

(11) MIM Software Inc.

(12) Minomic International Limited

(13) Philips Electronics Nederland BV

(14) ProteoMediX

(15) Watson Medical
